# A Formal Approach to the Selection by Minimum Error and Pattern Method for Sensor Data Loss Reduction in Unstable Wireless Sensor Network Communications

**DOI:** 10.3390/s17051092

**Published:** 2017-05-12

**Authors:** Changhwa Kim, DongHyun Shin

**Affiliations:** Department of Computer Science and Engineering, Gangneung-Wonju National University, 150, Namwonro, Heungeop-myeon, Wonju-si, Gangwon-do 26403, Korea; dhshin@cs.gwnu.ac.kr

**Keywords:** sensor data loss reduction, wireless sensor networks, underwater acoustic sensor networks, unstable communications, SMEP method, average sensor value error rate

## Abstract

There are wireless networks in which typically communications are unsafe. Most terrestrial wireless sensor networks belong to this category of networks. Another example of an unsafe communication network is an underwater acoustic sensor network (UWASN). In UWASNs in particular, communication failures occur frequently and the failure durations can range from seconds up to a few hours, days, or even weeks. These communication failures can cause data losses significant enough to seriously damage human life or property, depending on their application areas. In this paper, we propose a framework to reduce sensor data loss during communication failures and we present a formal approach to the Selection by Minimum Error and Pattern (SMEP) method that plays the most important role for the reduction in sensor data loss under the proposed framework. The SMEP method is compared with other methods to validate its effectiveness through experiments using real-field sensor data sets. Moreover, based on our experimental results and performance comparisons, the SMEP method has been validated to be better than others in terms of the average sensor data value error rate caused by sensor data loss.

## 1. Introduction

There are wireless networks in which communications are typically unsafe. Most wireless sensor networks belong to this category of networks. Usually, they have several shortcomings caused by limited resources, such as short-range and low-speed communications, limited-power batteries, low memory capacity, and low-speed processors [1,2,3,4,5]. In particular, their communications are vulnerable to environmental changes, such as foggy, rainy, hot, cold, or dry weather conditions, or location, among other factors [6,7,8,9,10,11,12]. Another example of an unsafe communication network is an underwater acoustic sensor network (UWASN). In underwater acoustic communication, there are several barriers that need to be overcome: low bandwidth, high propagation delay, high error rate, multi-path propagation, strong signal attenuation, and time-variant channels [13,14,15,16,17,18]. The time variation of channels in particular is one of primary causes of unsafe underwater communications. Underwater environmental changes, such as temperature, salinity, pressure, underwater fluid, bubbles, and noise, among others, are the main causes of time variations of channels [13,14,15,16,17,18], but many other factors also influence channel time variations. Owing to the time-variant channels, communication failures occur frequently and the failure time durations may range from seconds up to a few hours, days, or even weeks. After the failure has ended, in most cases the communication resumes, but during the communication failure time certain problems may occur. One of these is the loss of sensor data sampled during the failure. Although senor data can be saved temporally in routers in the form of packets during the course of routing, most or all of them are typically lost. Because the routing queue is not designed for long-time data saving, but rather for short-time saving to facilitate fast routing service, its capacity is insufficient to include many packets. Moreover, even sensor data packets saved in the router will be lost as the saving time increases and the number of new packets for routing increases more and more. On the other hand, no routing can be so much as attempted in a case in which the sensor node does not communicate with any neighbor node. Nonetheless, there are many cases in which it is very important for sensor nodes to save sensor data sampled during communication failure and to resume transmitting them when the failure is recovered. The saved sensor data can also be read out after the sensor node is retrieved by its operators. By doing this, we can detect several facts or forecast future trends such as following, using the sensor data saved during communication failures:

1. *Detection of accidents or events*: What accidents or events occurred?
2. *Main agents of accidents or events*: What or who brought about the accidents or events?
3. *Cause, time, or place of accidents or events*: Why, when, or where did the accidents or events occur?
4. *Progress of accidents or events*: How did the accidents or events proceed?
5. *Analysis of the cause of accidents or events*: What or who was the cause of accidents or events?
6. *Analysis of effects of accidents or events*: What or whom did the accidents or events have effects on and what were these effects?
7. *Prediction of future trends*: What will be changed by accidents or events, and how, when, where, or why will these changes occur?

For longer communication failure durations, in many applications it is more important for us to know some or all of the above. However, as the failure duration is longer, networks or nodes have higher probabilities of sensor data loss in the course of transmission [19,20]. Accordingly, long-period communication failures cause a large amount of sensor data loss on sensor networks and such sensor data loss may cause disadvantages for human beings. For example, long-term sensor data loss in an ocean-area contamination disaster impedes not only the cause and origin identification of the contamination but also the analysis of its dissemination progress and pollution area. Successively, such blockings prevent humankind from the prediction and prevention of the future disasters and their movements [19,20,21,22]. Ultimately, the sensor data lossless preservation for long-term communication failure in the previous disaster is certainly helpful for us to predict and prevent the same or similar future disasters, even though we fail in the real-time monitoring and controlling for the current disaster.

Insufficient memory capacity of a sensor node, however, makes it impossible to save the entire sensor data set lost during a long-term communication failure. Therefore, it is necessary to minimize the loss of the original sensor data during communication failure and to restore the lost sensor data nearly to the level of the original sensor data after the communication failure recovery. In other words, we need a method to reduce the sensor data loss caused by the memory capacity limit of the sensor node during communication failure. To date, only a few such methods, including *winavg* [23], *delta* [23], *CQP* [24], and 2*MC* [19,20] have been proposed. These methods use compression to replace two or more consecutive sensor data with an average value as a representative or to select a representative between two or three consecutive sensor data. All the sensor data apart from the representatives become lost in these methods. The remaining representatives are transmitted to the destination site, where the lost sensor data are recovered to these representatives (in *winavg*) or to the values interpolated using them. Most of these methods, however, do not show good performance in the average error rate as a measure of the data loss because these use their own very simple rules without consideration for performance measures such as that average error rate. The 2*MC* method shows much better performance than *winavg*, *delta*, and *CQP* methods in the sensor data loss minimization, because this method uses an error rate as a performance measure in selecting representatives. However, since the *2MC* method can fail to find the absolute positions of the lost sensor data, it has fatal drawback of being unable to recover the lost sensor data. Moreover, even though a communication framework for using these methods in wireless sensor networks is necessary, no framework for this has been presented.

Against this background, in this paper, we propose a framework to reduce sensor data loss during communication failures and we present a formal approach for the Selection by Minimum Error and Pattern (SMEP) method that plays the most important role in the reduction of sensor data loss in the proposed framework. The SMEP method uses a line interpolation error measure in selecting representatives to minimize errors in recovering lost sensor data. Compared with other methods using real-field sensor data sets, the experimental results show that the SMEP method has a much better average error rate within up to 87.5% sensor data loss (that is, 12.5% compression ratio). The SMEP method is also feasible, in that it can recover all lost sensor data.

The remainder of this paper is organized as follows. In Section 2, we introduce related works on technologies for sensor data compression, including *winavg* [23], *delta* [23], *CQP* [24], and 2*MC* [19,20], which are mostly applicable for sensor data loss reduction in sensor networks. Section 3 proposes a communication framework for the reduction of sensor data loss during communication failures. The proposed SMEP method can be applied to this framework, playing an important role. In Section 4, the fundamental formal definitions and properties of sensor data, compression interval, and round compression are introduced. These are necessary to introduce the SMEP method. Before presenting a detailed description of the SMEP method, the basic formal concepts and properties of the SMEP method are introduced in Section 5. Based on these, Section 6 formally presents the main data structures and examines their properties. These data structures and properties are used not only for compression in the SMEP method algorithm, but also for decompression and recovery in the destination node. Section 7 presents the SMEP algorithms necessary to compress the original sensor data during communication failures, which use the data structures and properties introduced in previous sections. The decompression and lost sensor data recovery from compressed sensor data by the SMEP algorithms is introduced in Section 8. For decompressing and recovering, the main algorithm in Section 8 uses the compressed sensor data and compression information in the data structures introduced in Section 6. A simple line interpolation algorithm is applied to the recovery of the lost sensor data between two consecutive compressed sensor data. In Section 9, we show the performance results and analysis in comparison with the four aforementioned sensor data compression methods, *winavg*, *delta*, *CQP*, and *2MC*, and we conclude this paper in Section 10.

## 2. Related Work

Many methods for sensor data reduction in wireless sensor networks have been proposed, mainly in three categories: data aggregation, data approximation, and query-based processing. The data aggregation methods cause intermediate or energy-intensive sensor nodes to merge into one packet several sensor data in packets transmitted in the course of routing [25,26,27]. Consequently, by reducing the number of transmissions, they have the effect of increasing energy efficiency and prolonging network life in wireless sensor networks [25,26,27].

The data approximation methods make sensor nodes transmit reduced sensor data, instead of transmitting all the original sensor data [28,29,30,31,32,33]. As a result, these methods also reduce the number of sensor data transmissions, similarly to data aggregation methods. Mostly, these methods use correlation or regression models between two or more different types of sensor data originated from different sensors. They transmit the representatives instead of all sensor data, but the untransmitted sensor data are restored to the approximate values using these correlation or regression models. The data approximation methods have overheads in that they inevitably need a priori knowledge, such as correlation or regression models, depending on the relationships among the sensor data [28,29,30,31,32,33].

Query-based processing is an on-demand sensor data transmission method. In this category, when each sensor node receives queries from monitoring and control stations, it transmits only the resulting sensor data corresponding to each query as its responses. Thereby, this method reduces the number of sensor data transmissions, avoiding constant transmission [34,35,36,37,38]. Additionally, query aggregation and results merging methods have been presented for avoiding redundancy in the response results [37,38].

Almost all the mechanisms in the above categories focus only on the energy efficiency resulting from reducing transmissions. Specifically, most of them are based on the premise that transmissions are in real-time and stable in communications. Therefore, in the cases of the absence of their recovery methods in these mechanisms, the sensor data sampled during a communication failure period are lost and the lost sensor data cannot be recovered [20,21,22,24].

A few mechanisms to recover the lost sensor data during transmission and routing have been proposed [39,40]. These mechanisms, however, require a priori knowledge obtained through preprocessing normal sensor data for detecting the locations of the lost sensor data and restoring them to their approximate values. Since these methods are based on real-time transmission, they too have the same problems described in the above three categories.

For the data loss reduction in unstable networks, delay/disruption tolerant network (DTN) techniques have been proposed [41,42,43,44]. These DTN techniques are asymmetric networking technologies that can be used in network environments where stable communication connections are not guaranteed. Generally, if sensor data were lost owing to network problems, it has been required to re-route from the source to the destination for its retransmission. Instead of doing this, these techniques preserve the transmission-intermitted sensor data packets in routers or intermediate nodes until these packets can be normally transmitted to the next node. In these methods, however, there high probabilities that, at the time of communication failure, bunches of sensor data packet streams gather at the specific router or intermediate node in a short period of time. Therefore, the routing queue capacity rapidly reaches its full state, and, thereafter, all the packets of sampled sensor data will be lost and not recovered, because of the absence of any compression and recovery method. Moreover, because packets with extra information such as headers or footers, and not bare data, are saved in routing queue space, the DTN techniques have high probabilities of more rapidly reaching the queue-full state, causing more data loss due to the inefficient use of memory space.

One of the scenarios for avoiding or resolving the problems discussed above is described as follows. Each sensor node is aware of communication failure. Thereafter, it saves its sampled sensor data to its own memory space without depending on other nodes like routers or intermediate nodes, and compresses them into the same memory whenever its memory space researches full state, until the communication failure is recovered. As soon as the sensor node is aware of communication failure recovery, the node starts transmitting the saved original or compressed sensor data to the destination and then, after receiving them, the destination decompresses and recovers the original and lost sensor data. This is the scenario that this paper is pursuing.

A framework relevant to the above scenario has been proposed in our previous work [21]. The conceptualized overview version on this framework is presented shortly in Section 3 of this paper. Meanwhile, the compression and recovery mechanisms capable of following our scenario have been presented in a small number of works in the literature. The *winavg* and *delta* methods are two of them and they have been introduced in [23]. Firstly, the *winavg* method [23] calculates an average value for a given number of sensor data (that is, window size) among sensor data in the queue-full sate and saves this average value as the representative value of the sensor data in the window size. This method recovers all the lost sensor data within each window to the same unified values as the average value of the window. Accordingly, the bigger the window size and the standard deviation of the sensor data within each window, the larger the error size between the original sensor data and its recovered data is. The *delta* method, which is also very briefly introduced in [23], selects from two sensor data adjacent to the last selected sensor data, the one of which the value difference from the last selected sensor data is bigger than the other. Thus, the unselected sensor data between these two sensor data is discarded. The *delta* method recovers the sensor data lost between these two consecutive sensor data to the selected one. These two methods have advantages to restore the lost sensor data to the approximated values instead of losing all of them after the data queue becomes full. However, they tend to increase the error, radically, if queue overflows by communication failures last for a long time or if sensor data values change irregularly with high deviation. The Circular Queue based on Period Compression *(CQP)* method [24] is also a simple method, and one of our previous works, which is capable of following our scenario to reduce sensor data loss. The *CQP* method is similar to but simpler than the *delta* method. The *CQP* method selects unconditionally the first sensor data at any time of the queue overflow. Then, it selects unconditionally the second sensor data between two sensor data located just next to the last selected sensor data in the sensor data sequence in the queue, using a circular queue. In spite of the simplest method, this method shows similar or better performance in the recovery of the lost sensor data compared with the *winavg* and *delta* methods. The *2MC* method, another one among our previous works, has been presented in [19,20]. In the compression process, this method divides a sensor data sequence into three-point (sensor data) intervals and the last sensor data of each interval is shared with the first sensor data of its next interval. For each odd-numbered compression interval, it selects either a minimum-valued sensor data, if the sensor data shows a decreasing trend in the interval, or a maximum-valued sensor data, if they show an increasing trend. For each even-numbered compression interval, it selects the sensor data with minimum value difference from the sensor data value selected in the neighboring odd-numbered compression interval. Compared with the *winavg*, *delta*, and *CQP* methods, this method shows the best performance in the average error rate [19,20]. Recently, however, our team has found out its serious problems, that it is possible to select two sensor data for a compression interval and, in that case, the positions of all the sensor data after these sensor data are miscalculated. For this reason, the *2MC* method is infeasible in the decompression and recovery of the compressed and lost sensor data. In fact, the main reason for such an infeasibility is that the *2MC* method is just an idea-level method and lacks a mathematical basis. The *2MC* method also selects, after the *n*th compression, only the $2^{n}$th sensor data among the $2^{n}$ next original sensor data and discards $2^{n}$-1 sensor data. Therefore, there has remained in the *2MC* method room for performance improvement by saving all the new original sensor data sampled after each compression, so that they cannot be lost until the next compression. The main reason why this paper proposes the SMEP method is to resolve these problems through a mathematical approach and show its feasibility.

## 3. Communication Framework Overview for Sensor Data Loss Reduction

In this section, we present an overall framework to reduce sensor data loss in the course of communication failure. In fact, this framework provides for the requirement analysts or designers a guideline for the identification and realization of functionalities necessary to support the reduction of sensor data loss during communication failures in wireless sensor networks.

**Figure 1:**
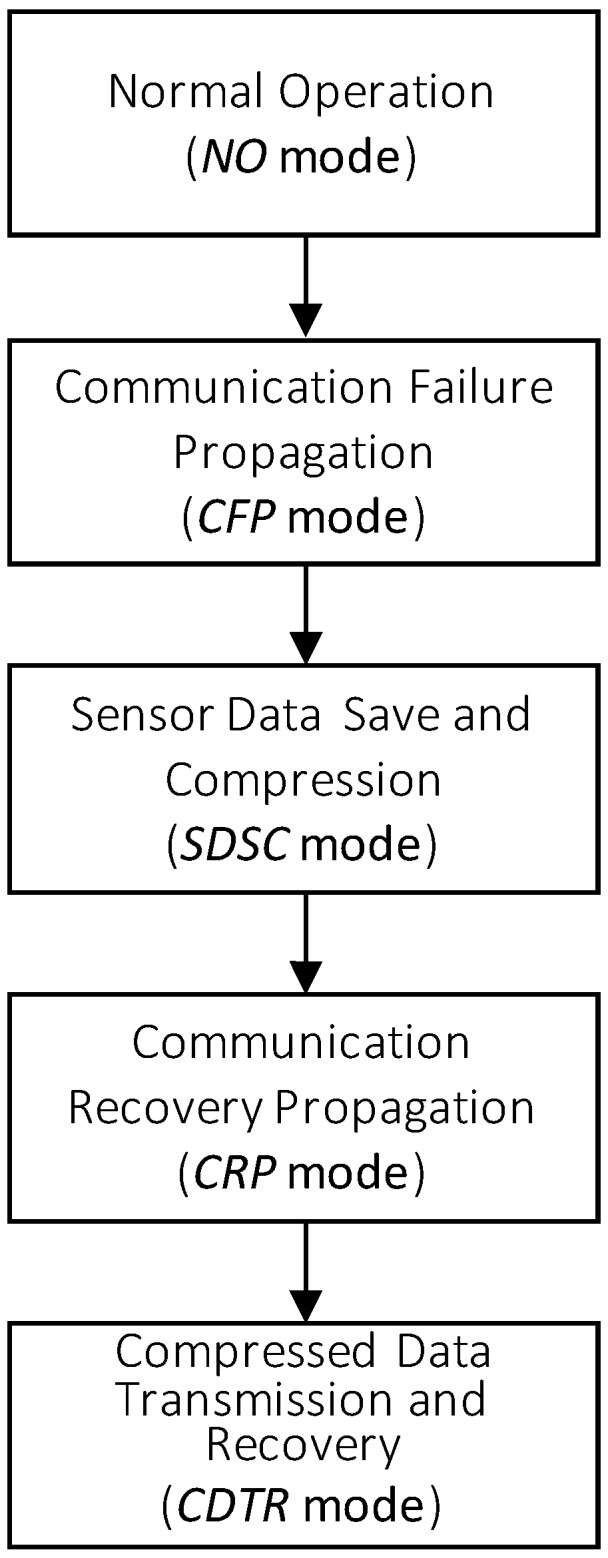
A communication framework for sensor data loss reduction.

Our overall framework has five sequential modes over a sensor node, as shown in Figure 1, which are the *Normal Operation* (*NO*), *Communication Failure Propagation* (*CFP*), *Sensor Data Save and Compression* (*SDSC*), *Communication Recovery Propagation* (*CRP*), and *Compressed Data Transmission and Recovery* (*CDTR*) modes,. The detailed explanation of the overall framework is as follows:

In the *NO* mode, each sensor node in a sensor network performs its operations normally, such as sampling, processing, sending, or receiving sensor data. There occurs no communication failure in this mode. When a communication failure occurs, the sensor network mode moves from the *NO* mode to the *CFP* mode. In this mode, the first sensor node to detect a communication failure propagates to its neighbor nodes a Communication Failure (*CF*) message to notify them of the communication failure occurrence. Every sensor node to receive a *CF* message tries to find an alternative routing path, other than via the sender node of the *CF* message. If the sensor node finds a new routing path, it invalidates the routing path via the sender node of the *CF* message and performs transmission operations through the new routing path. If any new routing path is not found, then the sensor node propagates to its neighbor nodes the Communication Failure (*CF*) message and its mode moves into *SDSC* mode. In this mode, the sensor node starts saving sensor data generated from its sensor into its data queue. The sensor node performs the compression operation over some or all of the sensor data in the queue when the data queue is full. In this mode, as soon as the sensor node receives from the previous sensor node of the CF message a Communication Recovery (*CR*) message to notify that the previous communication failure has been recovered, the sensor node mode moves from the *SDSC* mode into the *CRP* mode. In *CRP* mode, the sensor node validates the invalidated routing path via the sender, propagates the CR message to its neighbor nodes again and then its mode moves to the *CDTR* mode. In the *CDTR* mode, the sensor node reverts to using the validated or recovered routing path from the previous mode as a new primary routing path. In this mode, the sensor node sends all the saved or compressed sensor data in data queue and the compression information through the recovered routing path. After that, the sensor node mode returns to the *NO* mode and proceeds with normal operations. The destination node to receive all the compressed sensor data and the compression information proceeds with restoring all the lost sensor data from the compression in *SDSC* mode to the approximated values, which are similar to their original sensor data.

There occurs sensor data loss due to the compression operations in *SDSC* mode. In fact, sensor data loss is inevitable in the communication failures with no alternative routing path. Specifically, in the long-term communication failures, without taking any action after the full state of the data or the routing queue, almost all of the new generated and routed sensor data will be lost inevitably. Therefore, lost sensor data cannot be restored to the same values as the original sensor data, or even to similar values. The only solution to resolve this problem is to devise a compression method to compress sensor data in a full queue state and restore the lost ones in the compression process to the same or similar values. For this reason, the compression in the *SDSC* mode is the most important task in our framework to reduce sensor data loss and the *SMEP* method presented from Section 4 to Section 8 is one of such compression methods.

## 4. Basic Definitions and Properties

Fundamental formal definitions and properties are presented in this section. These are necessary to introduce the SMEP method in Section 5, Section 6 and Section 7, and the decompression and recovery algorithms in Section 8.

**Definition** **1.** The sensor data sequence S is a list of periodic sensor data, S_0_, S_1_,…, S_n−1_ such that for each i (0 ≤ i ≤ n − 1) S_i_ is a sensor data and if j < k (0 ≤ j, k ≤ n − 1), then S_j_ is a sensor data generated before the sensor data S_k_. We represent the sensor data sequence as S = S_0_, S_1_,…, S_n−1_.

We call the number of sensor data in the sensor data sequence *S* the *size* of *S* or the *length* of *S* and represent it with |*S*|.

For simplicity, let us define terms, *ground sequence* and *base sequence*.

**Definition** **2.** *The ground sequence* or *ground sensor data sequence is defined as a sensor data sequence of which each sensor data does not pass through any compression after its generation from a sensor.*

**Definition** **3.** The base sequence is defined as a sensor data sequence of which each sensor data is selected through the same compressions and is a target for another compression.

**Definition** **4.** Given a sensor data sequence, S = S_*0*_, S_*1*_,…, S_n−*1*_, a compression interval I with the size m with respect to S is defined as a part of S, i.e., I = S_i_, S_i+*1*_,…, S_i+m_, which consists of m + 1 consecutive sensor data in S and is a target for compression.

For simplicity, we represent a compression interval *I* with size *m*, *S_i_*, *S_i+_*_1_,…, *S_i+m_* as [*i*, *i + m*] by using only the first and last subscripts of its *m + 1* consecutive sensor data . We can know easily that if *I* = [*i*, *j*] is a compression interval, then the size of *I* is *j* − *i*. The compression interval is a sensor data sequence, too. However, note that the size of the compression interval *I* is different from the size of the sensor data sequence *I*. In fact, the size of the compression interval *I* is 1 less than the size of a sensor data sequence *I*. A compression on the compression internal *I* = [*i*, *i + m*] is an operation to select one or more sensor data from all sensor data in *I* and discard all other sensor data except the selected ones.

**Definition** **5.** Consider two compression intervals I = [i, j] and J = [k, l] with respect to a sensor data sequence S. Then, if j = k or l = i, I and J are defined as consecutive compression intervals in S. If each size of two consecutive compression intervals is the same as m then we call that as consecutive compression intervals with size m.

According to this definition, if two compression intervals are consecutive then the last sensor data of one of two compression intervals is the same as the first sensor data of the other.

**Theorem** **1.** Let I = [i, j] and J = [k, l] be consecutive compression intervals with size m and n, respectively, in a sensor data sequence S. If i < k or i < l or j < l, then J = [i, l] is a compression interval with the size m+n with respect to S (For the proof refer to Appendix A).

Given two consecutive compression intervals, *I* = [*i*, *j*] and *J* = [*k*, *l*], of a sensor data sequence, if *i* < *k* or *j* < *l* (this is, *j* = *k*)*,* then we represent it with *I* < *J* and we call that *I* is smaller than *J* or that *J* is greater than *I*.

**Definition** **6.** Suppose that I = [i, j] and J = [k, l] are consecutive compression intervals with respect to a sensor data sequence, S, and I < J. We define the merging operation ● of I and J as I ● J = [i, l] and we call I ● J the merged compression interval of I and J.

We can prove easily the following theorem, an association law of the merging operation.

**Theorem** **2.** Let I, J, and K be consecutive compression intervals and I < J < K. Then, the merging operation satisfies the associative low. That is, (I ● J) ● K = I ● (J ● K).

**Definition** **7.** Consider consecutive compression intervals, I_*0*_, I_*1*_,…, I_m−*1*_ with respect to a sensor data sequence, S, where I_*0*_ < I_*1*_ < I_*2*_ < … < I_m−*1*_. If S = I_*0*_ ● I_*1*_ ● I_*2*_ ● … ● I_m−*1*_, then a set C_S_ = (I_*0*_, I_*1*_, I_*2*_,…, I_m−*1*_) is called as a compression interval covering on S. When the size of every compression interval in the compression interval covering C_S_ is the same as k, we call C_S_ the k-size compression interval covering.

From definitions, we can get the relationship between a sensor data sequence and its 2-size compression interval covering as the below. For the proof refer to Appendix B.

**Theorem** **3.** Let S and C_S_ = (I_*0*_, I_*1*_, I_*2*_,…, I_m−*1*_) be a sensor sequence and a 2-size compression interval covering on S, respectively. Then, the following propositions are true:

**Proposition** **1.** |S| = 2m + 1.

**Proposition** **2.** Given a compression interval, I_i_ = S_p_, S_q_, S_r_, for some integer i, 0 ≤ i ≤ m − 1, positions of S_p_, S_q_, and S_r_, is 2i, 2i + 1, and 2i + 2, respectively, in S.

In the *SDSC* mode already introduced in Section 3, in order for a sensor node not to loss sensor data generated periodically from its sensor, the sensor node has to have its own data queue to save lost sensor data in the *SDSC* mode. However, whenever the data queue is full in the *SDSC* mode, the *2MC* method executes a compression on a sensor data sequence of its data queue. Now, let us define a *compression round* as follows:

**Definition** **8.** A round compression is an operation defined as follows and a compression round is the number of the compression executions:

(i) A ground sequence is in the *0-th round compression*.
(ii) The *i*-th *round compression* is a compression executed when *S* is a sensor data sequence corresponding to a full data queue and each sensor data in *S* have been derived through the *i-1* th compression.

At the time when a compression is executed, some of sensor data sequence are selected and the others are discarded. Here, the selected become the compressed sensor data but the discarded becomes the lost sensor data. In order for each of lost sensor data to be restored, its position should be known or found. The SMEP method uses position information about the selected sensor data to find positions the sensor data lost in compression.

Besides the above definitions more definitions are introduced in the following sections whenever necessary. All of main notations and terms are listed in Table 1 with their definition numbers. Refer to definitions if necessary.

## 5. Basic Concepts for the SMEP Method

The SMEP method uses two selection rules for two consecutive compression intervals to cover a sensor data sequence for the compression; *selection by compression interval pattern* and *selection by minimum error*. This section presents basic SMEP method concepts helpful to understand its data structure elements and algorithms introduced in Section 6, Section 7 and Section 8 in detail, including two selection rules.

### 5.1. SMEP Compression Process Overview

The SMEP proceeds the compression with 2-size consecutive compression interval covering with respect to a sensor data sequence with the size *2^m^ + 1* for in integer *m*, *m ≥ 1*. Actually, the size *2^m^ + 1* or *2^m^* is the same as the length of a part of data queue used for saving and compressing the sensor data generated from a sensor in the *SDSC* mode and the sensor data sequence for the compression is a sequence of sensor data in the queue. Depending on the *2^m^ + 1* size, we get the below theorem (For the proof refer to Appendix C):

**Theorem** **4.** Let S and C_s_ be a sensor data sequence S and a 2-size compression interval covering of S and C_s_ = (I_*0*_, I_*1*_,…, I_l_), respectively. If the size of S is 2^m^ + 1 for an integer m ≥ 1, then l = 2^m−*1*^ − 1, that is, the number of compression intervals of C_s_ is 2^m−*1*^.

**Figure 2:**
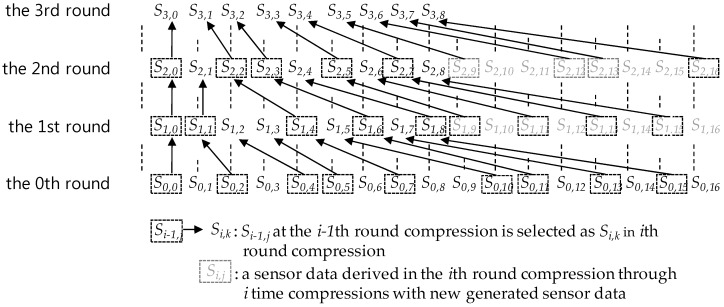
Sensor data selection in the 1st, 2nd and 3rd round compressions.

**Figure 3:**
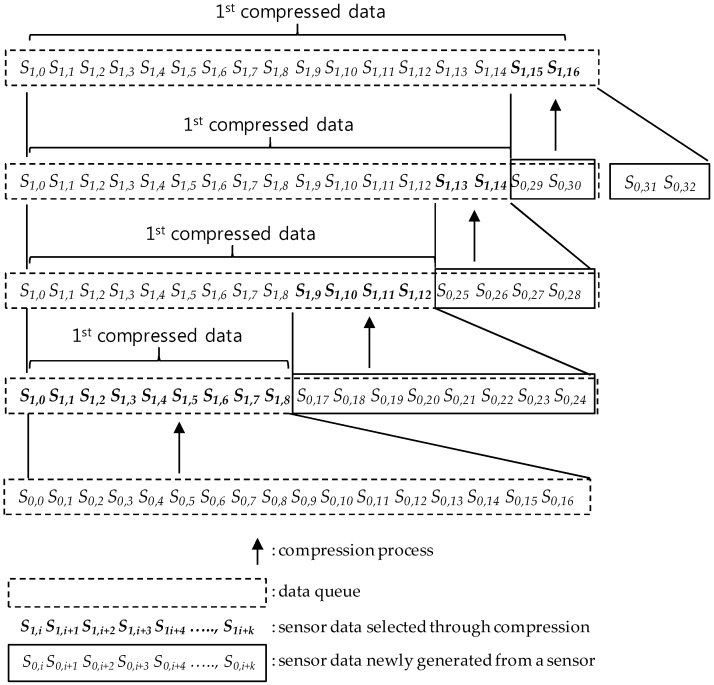
The first round compression process.

Whenever a compression round is proceeded, one sensor data in each compression interval is selected and the others are discarded in it. At this time, the discarded sensor data becomes the lost sensor data. Figure 2 and Figure 3 show the compression process on a sensor data sequence in a data queue with the size 17 = 2^4^ + 1. As shown in Figure 2, in the *c*-th (*c* = 1, 2, 3,…) round compression, sensor data after the c-1 th round compression are selected as follows:

At first, the first sensor data *S_c-_*_1*,*0_ is selected as *S_c,_*_0_, unconditionally, where *S_c,i_* means the *i*-th sensor data of a sensor data sequence derived through the *c*-th round compression. Then, by selecting one sensor data per every compression interval, according to the order between compression intervals in the 2-size compression interval covering, the compression derives a new compressed sensor data sequence. One of both of the 2nd and the 3rd sensor data in each interval is selected so that the SMEP method should keep a selection rule, *one per compression interval*, for all compression intervals except the 1st compression interval. In selection, the *i*-th sensor data selected in the previous compression round becomes the *i*-th sensor data in the new compressed sensor data sequence. After the compression, the size of the new sensor data sequence shrinks to the half the previous one, this is, *2^m−^*^1^ *+* 1, occupying data queue space with the same size, and the *2^m−^*^1^ *+* 1 size queue space remains empty. After that, as sensor data generated newly from a sensor are inserted into the remaining queue space, the space becomes full like Figure 2 and Figure 3 so that new sensor data should be compressed with the same way until all sensor data in the full state data queue are derived through c time compressions.

When all sensor data of a sensor data sequence in the full state data queue are derived through c time compressions, the *c*-th round compression has been completed and the new *c+1* th round compression begins. Figure 3 shows only the first round compression process.

### 5.2. I-bit Position and Generation

**Figure 4:**
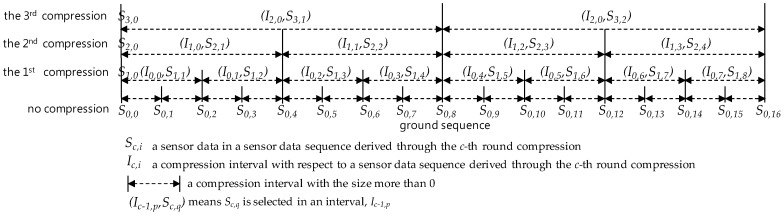
A sensor data selection in the merging of a pair of two compression intervals.

Figure 4 shows the compression process in the aspect of the merging of two intervals. Compression is conducted by selecting just one from sensor data in a compression interval that corresponds to the merging of two sequent compression intervals of the base sequence. For example, in Figure 4, *S*_1,3_, a sensor data, is selected as the one from a compression interval, *I*_0,2_ = [4, 6]_0_. Here, *I_c,i_* means the *i*-th position compression interval in the 2-size compression interval covering on a sensor data sequence derived though the *c*-th round compression. Additionally, [*p*, *q*]*_c_* represents an compression interval, [*p*, *q*] on a sequence derived through the *c*-th round compression. Shortly, *(I*_0,2_*, S*_1,3_*)* in the figure means the sensor data *S*_1,3_ is selected in the compression interval *I*_0,2_.

For further description, we define the term *cover*:

**Definition** **9.** Let S_i_ and I be a sensor data and a compression interval with respect to a sensor data sequence, respectively. S_i_ covers I if and only if S_i_ is selected in I.

In Figure 4, *S*_2,2_ covers *I*_1,1_ and it is selected one of *S*_1,3_ and *S*_1,4_ to cover *I*_0,2_ and *I*_0,3_, respectively. Note that *S*_2,2_ covers *I*_0,2_●*I*_0,3_ in this figure. We define the term *cover* on the view of compression interval:

**Definition** **10.** Given two compression intervals I_c-*2*,j_ and I_c-*2*,j+*1*_ and a selected sensor data S_c,k_ to cover I_c−*1*,k_ for c ≥ 2, I_c−1,k_ covers I_c−*2*,j_ ●I_c−*2*,j+*1*_ if and only if S_c,k_ covers I_c−*2*,j_ ●I_c−*2*,j+1_.

According to the definition, *I*_1,1_ covers *I*_0*,*2_●*I*_0,3_ in Figure 4. Moreover, *I*_2*,*0_ covers *I*_1*,*0_●*I*_1*,*1_ and *I*_1*,*0_ and *I*_1*,*1_ also cover *I*_0*,*0_●*I*_0*,*1_ and *I*_0*,*2_●*I*_0*,*3_. Since *S*_3*,*1_ covers *I*_2,0_ and *S*_3,1_ covers *I*_0,0_●*I*_0,1_●*I*_0,2_●*I*_0*,*3_ *I*_2,0_, *I*_2,0_ also covers *I*_0*,*0_●*I*_0*,*1_●*I*_0*,*2_●*I*_0*,*3_ *I*_2*,*0_. For the generalization of this property, we introduce the below Lemma 1, Lemma 2 and Theorem 5. For their proofs refer to Appendix H, Appendix I, and Appendix D, respectively.

**Lemma** **1.** Let S and S^c^ = S_c,*0*_, S_c,*1*_, …, S _c,*2*_^m^ for integer m ≥ 0 be a ground sequence and a sensor data sequence derived through the c-th round compression on S, respectively. Let C_s_^c^ = (I_c,*0*_, I_c,*1*,_ …, I_c,*2*_^m−*1*^_−*1*_) be a 2-size compression interval covering. Then, in the SMEP method, if for k ≥ 0, I_c,k_ covers I_c−*1,2*k_ ●I_c−*1,2*k+*1*_.

**Lemma** **2.** Let S and S^c^ = S_c,*0*_, S_c,*1*_, …, S _c,*2*_^m^ for integer m ≥ 0 be a ground sequence and a sensor data sequence derived through the c-th round compression on S, respectively. Let C_s_^c^ = (I_c,*0*_, I_c,*1*,_ …, I_c,*2*_^m−*1*^_−*1*_) be a 2-size compression interval covering on S^c^. Then, in the SMEP method, if for i ≥ 0 and j ≥ 1 S_c,i_ covers I_c−*1*,j_ then j = i − 1 and I_c−1,j_ covers I_c−*2,2*(i−*1*)_ ●I_c−*2,2*i−*1*_.

**Theorem** **5.** Let S and S^c^ = S_c,*0*_, S_c,*1*_, …, S _c,*2*_^m^ for integer m ≥ 0 be a ground sequence and a sensor data sequence derived through the c-th round compression, respectively, based on S. Then, in the SMEP method, for integers c ≥ 1 and for any integer i ≥ 1, S_c,i_ covers I_0,(i−*1*)*2*_^c−*1*^ ● I_0,(i−*1*)*2*_^c−*1*^_+*1*_ ● ⋅⋅⋅ ● I_0,i__⋅*2*_^c−*1*^_−*1*_.

This theorem tells S_c,i_ is the sensor data selected in *I*_0*,(i−*1*)*2_*^c−^*^1^ *● I*_0*,(i−*1*)*2_*^c−^*^1^*_+_*_1_ *●* *⋅⋅⋅* *● I*_0*,i*_*_⋅_*_2_*^c−^*^1^*_−_*_1_*.* By the way, since *I*_0*,k*_ *= [2k, 2k + 2]*_0_ and *I*_0*,(i−*1*)*2_*^c−^*^1^ *● I*_0*,(i−*1*)*2_*^c−^*^1^*_+_*_1_ *●* *⋅⋅⋅* *● I*_0*,i*_*_⋅_*_2_*^c−^*^1^*_−_*_1_ *= [2(I − 1)2^c−*1*^, 2(i − 1)2^c−*1*^ + 2]*_0_ *● [2(i − 1)2^c−*1*^ + 2, 2(i − 1)2^c−*1*^+4]*_0_ *●* *⋅⋅⋅* *● [i2^c^ − 2), i2^c^]*_0_ *= [(i − 1)2^c^, i2^c^]*_0_, *S_c,i_* is the sensor data selected in the compression interval *[(i−1)2^c^, i2^c^]*_0_*.*

**Corollary.**  Let S_c,i_ be the i-th position sensor data in a sensor data sequence derived through the i-th round compression. Then, the position of S_c,i_ exists in [(I − 1)2^c^, i2^c^]_0_.

As described already before, the positions of the selected data should be necessarily known in order that each of all lost sensor data is restored. The very function that lets the SMEP method know where each sensor data is either selected or lost is I-bit position.

**Definition** **11.** I-bit position is defined recursively as a function to generate the bit string about the position of S_c,i_ in a compression interval according to the following rules:

*Rule 1. For any c = 1, 2,…, n, I-bit position(*$S_{c,0}$ *) is not defined.*

*Rule 2. In 1-th round compression, for each I*_0*,k*_ *in* $C_{S^{0}}$ *for k = 0, 1, …,* $\left|C_{S^{0}}\right|-1$ *and for S*_1*,i*_ *selected from I*_0*,i−*1_ *= [2i − 2, 2i]*_0_ *= S*_0*,*2*i−*2_*, S*_0*,*2*i−*1_*, S*_0*,*2*i*_*,*

$$\text{I-bit position}\left(S_{1,i}\right)=\{\begin{array}{c} 0\;if\;S_{0,2i-1}\;is\;selected\;as\;S_{1,i}\;\\ 1\;if\;S_{0,2i}\;is\;selected\;as\;S_{1,i}\; \end{array}$$

*Rule 3. In c-th round compression, for each I_c−_*_1*,k*_ *in* $C_{S^{c-1}}$ *for k = 0, 1, …,* $\left|C_{S^{c-1}}\right|-1$ *and for S_c,i_ selected from I_c−_*_1*,i−*1_ *=* *[2i − 2, 2i]_c−_*_1_ *= S_c−_*_1*,*2*i−*2_*, S_c−_*_1*,*2*i−*1_*, S_c−_*_1*,*2*i*_*,*

$$\text{I-bit position}\left(S_{c,i}\right)=\{\begin{array}{c} 0\;\Theta I-bit\;position\left(S_{c-1,2i-1}\right)\;if\;S_{c-1,2i-1}\;is\;selected\;as\;S_{c,i}\;\\ 1\;\Theta I-bit\;position\left(S_{c-1,2i-1}\right)\;if\;S_{c-1,2i}\;is\;selected\;as\;S_{c,i}\; \end{array}$$

**Figure 5:**
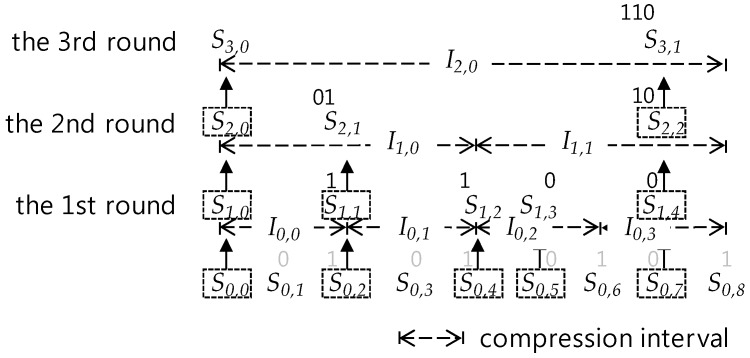
I-bit position generation process of sensor data in compression interval.

Shortly, we call I-bit position of *S_c,i_*_-th_e value of *I-bit position(S_c,i_).* The I-bit position represents the relative position of a sensor data within a compression interval. In fact, given an *I-bit position(S_c,i_)*, the real relative position of *S_c,i_* in its interval is *(the value of I-bit position(S_c,i_))* + 1, As compression makes progress, two consecutive compression intervals that two competitive sensor data for selection cover is merged into one compression interval covered by the selected sensor data. The above definition lets us know how the position of a selected sensor data in a merged compression interval is determined by positions of two competitive sensor data. Figure 5 illustrates such a process to generate the I-bit position of a selected sensor data in compression. In the 1st round compression, *S*_0,0_, *S*_0,2_, *S*_0,4_, *S*_0,5_, and *S*_0,7_ are selected as *S*_1,0_, *S*_1,1_, *S*_1,2_, *S*_1,3_, and *S*_1,4_ from compression intervals, *I*_0,0_, *I*_0,1_, *I*_0,2_, and *I*_0*,*3_, respectively. Here, note that *S*_0*,*0_ is unconditionally selected as *S*_1,0_ and its I-bit position is undefined according the definition *Rule 1*, and *S*_0*,*2_ is selected as *S*_1,1_ in the compression interval *I*_0,0_. According to the *Rule 2* of the above definition, their corresponding I-bit positions are *null* (i.e., undefined), 1, 1, 0, and 0 in the 1st round compression. As in the 2nd round compression, *I*_0,0_ and *I*_0,1_, are merged into *I*_1*,*0_ and *I*_0*,*2_ and *I*_0*,*3_ are also merged into *I*_1,1_, *S*_2,0_, *S*_2,1_ and *S*_2,2_ are selected from *S*_1,0_, *S*_1,1_, and *S*_1*,*4_, respectively. Each of them is selected as one per each compression interval after the 1^st^ compression, except the unconditionally selected *S*_2,0_ in *I*_1,0_. *S*_1,0_ and *S*_1*,*1_ are positioned in the first compression interval *I*_1,0_ and *S*_1,4_ is positioned in the second one *I*_1*,*1_. According to the definition *Rule 1*, I-bit positions of *S*_2,0_ is undefined as *null*, again. In addition, by applying the first condition of the definition *Rule 3* to the first compression interval, I-bit position of *S*_2*,*1_ are created by adding 0 bit to the front of the *I-bit position of S*_1,1_ and, ultimately, they become to be 01 as a relative position bit string for the merged compression interval *I*_2,0_. Meanwhile, *S*_2,2_ selected from *S*_1,4_ exists in the second compression interval *I*_1,1_ among two consecutive compression intervals covered by *I*_2,0_. In this case, we have to apply the second condition of the *Rule 3* and so we can get the bit string 10 by adding the bit 1 to the front of I-bit position of *S*_1,4_. After that, *S*_3,0_ and *S*_3,1_ are selected from *S*_2,0_ and *S*_2,2_ located in the first and second compression intervals *I*_1,0_ and *I*_1,1_ covered by *I*_2,0_. Therefore, we can apply the *Rule 1* and the condition of the *Rule 3* to the generation of I-bit positions of *S*_3,0_ and *S*_3,1_ so to get *null* and 110, respectively.

As a new round compression proceeds, the length of I-bit position of a selected sensor data makes one more increase in the new round compression than in the previous round compression, resulting to the c length bit string for the *c*-th compression, ultimately.

### 5.3. Absolute Position

As soon as a sensor data is selected during a compression, the SMEP method generates an I-bit position corresponding to the sensor data and updates *I-bit position sequence* defined in the below, appending its I-bit position to it:

**Definition** **12.** Let S^0^ and S^c^ = S_c,0_, S_c,1_, …, S_c,n_ be a ground sequence and a sensor data sequence compressed from S^0^ through the c-th round compression. I-bit position sequence of S^c^ is defined as a bit string sequence that consists of concatenations of I-bit positions of all sensor data except S_c,0_ in S^c^. That is,

*I-bit position sequence of S^c^ = I − bit position(S_c,1_)* Θ *I-bit position(S_c,2_)* Θ… Θ *I-bit position(S_c,n_).*

Moreover, the position of the first bit of the I-bit position sequence is defined as 0. From the above definition, note that the I-bit position corresponding to the first sensor data is excluded from I-bit position sequence. In fact, we can remove it because the position of the first sensor data is always fixed as the first for every compression. In Figure 5, I-bit position sequence of *S*^1^ is 1100 (= 1 Θ 1 Θ 0 Θ 0) generated by concatenating I-bit positions of *S*_1,1_, *S*_12_, *S*_1,3_, and *S*_1,4_ except *S*_1,0_. I-bit position sequences of *S*^1^, and *S*^2^ are 0110 (=01Θ10), and 110 by the same way, respectively.

Given an I-bit position sequence, *B_p_*, of *S^c^*, for any sensor data, *S_c,k_*, we can get easily the *I-bit position* of *S_c,k_* by taking a substring from the *c(k − 1)* th position bit to *ck − 1* th position bit in *B_p_*. So, let us define a function, *BitSubstring*, now:

**Definition** **13.** Given an I-bit position sequence, B_p_, of S^c^, for any sensor data, S_c,k_, BitSubstring is a function to take a bit substring from the c(k − 1) th bit to ck − 1 th bit in B_p_, this is, BitSubstring(B_p_, c, k) = a bit substring from the c(k − 1) th bit to ck − 1 th bit in B_p_.

**Lemma** **3.** Given an I-bit position sequence, B_p_, of S^c^, for any sensor data, S_c,k_,

I-bit position(S_c,k_) = BitSubsring(B_p_, c, k) (For the proof refer to Appendix J). On the other hand, all data in S^c^ originate from S°. In fact, they are sensor data that compete to survive through selections in c time compressions. In order to restore the lost data in competences, for each sensor data in the compressed data sequence its position in the ground sequence, which we call the position the absolute position defined the below, has to be known:

**Definition** **14.** Let S_c,k_ be a sensor data that originates from S_0,i_. Then, the absolute position of S_c,k_ is defined as the position i of S_0,i_ in S°.

The absolute position of a sensor data after c compressions can be known with its I-bit position by the following Lemma (For the proof refer to Appendix K):

**Lemma** **4.** *Let S^0^ and S^c^ = S_c,0_, S_c,1_, …, S_c,n_ be a ground sequence and a sensor data sequence derived from S° through the c-th round compression, respectively. Given B_p_, an I-bit position sequence, for any S_c,k_ originated from S_0,i_ in S_0_, the absolute position i is computed as follows:*

(i) *i* = *0*, if *k* = *0*
(ii) *i* = *(k−1)2^c^* + *(I-bit position(S_c,k_))*_10_ + 1, if *k* ≥ *1*

By the two above lemmas, the following theorem is trivially true:

**Theorem** **6.** Given B_p_, an I-bit position sequence of S^c^, for any S_c,k_ (k ≥ 1), the absolute position of S_c,k_

*i = (k − 1)2^c^ + (BitSubstring(B_p_, c, k))*_10_ *+ 1, if k ≥ 1*

The above theorem is important in that it means that we can find the absolute position of a compressed sensor data if we know the I-bit position sequence, the number of compressions and the position of the compressed sensor data in the compressed sensor data sequence.

### 5.4. Selection by Compression Interval Pattern Rule

For a compression interval, *I_c_* = *[i, I + 2]_c_* = *S_c,i_*, *S_c,i+_*_1_, *S_c,i+_*_2_ with size 2, we can have one of nine cases depending on these sensor data values as follows:

*Case 1.* (Pattern 1) *S_c,i_* > *S_c,i+1_* > *S_c,i+2_*
*Case 2.* (Pattern 2) *S_c,i_* < *S_c,i+1_* < *S_c,i+2_*
*Case 3.* (Pattern 3) *S_c,i_* = *S_c,i+1_* = *S_c,i+2_*
*Case 4.* (Pattern 4) *S_c,i_* > *S_c,i+1_* < *S_c,i+2_*
*Case 5.* (Pattern 5) *S_c,i_* < *S_c,i+1_* > *S_c,i+2_*
*Case 6.* (Pattern 6) *S_c,i_* > *S_c,i+1_* = *S_c,i+2_*
*Case 7.* (Pattern 7) *S_c,i_* < *S_c,i+1_* = *S_c,i+2_*
*Case 8.* (Pattern 8) *S_c,i_* = *S_c,i+1_* > *S_c,i+2_*
*Case 9.* (Pattern 9) *S_c,i_* = *S_c,i+1_* < *S_c,i+2_*

**Figure 6:**
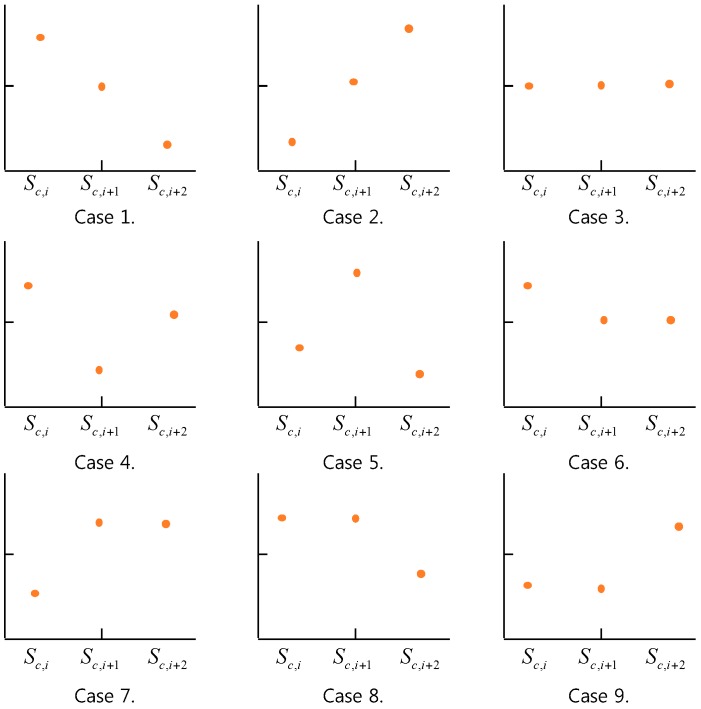
Nine patterns of sensor data in 2-size compression interval.

Each of the above cases shows its own pattern for *S_i_*, *S_i+_*_1_, *S_i+_*_2_ as shown in Figure 6.

Assume that *S^c^* is a sensor data sequence in a data queue for compression and $C_{S^{c}}$ = *(I_c_,*_1_, *I_c_,*_2_,…, *I_c_,_m_*) is a *2-*size compression interval covering with respect to *S^c^*. Then, we define the *selection by compression interval pattern* as follows:

**Definition** **15.** *Given a compression interval I_c,i_ = S_c,i_, S_c,i+_*_1_ *, S_c,i+_*_2_ *in* $C_{S^{c}}$ *with respect to Sc, the selection by compression interval pattern with respect to Ic,i is defined as the selection of a sensor data among Sc,i, Sc,i+1, Sc,i+2 according to the following selection by pattern rule:*

Selection by Pattern Rule: If Pattern 1 or Pattern 2 or Pattern 3 is true among nine patterns, then S_c,i+*2*_ is selected, otherwise S_c,i+*1*_ is selected.

Simply, we will call the *selection by compression interval pattern* rule the *pattern selection* rule or *selection by pattern* rule. The *selection by pattern* rule is derived not from noble theoretical results to minimize the errors between every lost sensor data and its corresponding original sensor data in the several compressions, but from empirical results through several experiments with several sets of real-field sensor data. Thus, the compression to use only the *pattern selection* method tends occasionally to show worse performance than the *CQP* method and others. For this reason, we have devises a method mixed up with the *pattern selection* method and the *selection by minimum error* method in Section 5.5.

On the other hand, in two consecutive compression intervals of 2-size compression interval covering, the first sensor data in the second interval is the same one with the third sensor data in the first interval according to Definition 5 in Section 4. If the third sensor data is selected in the first interval, the first sensor data cannot be selected in the second interval. For this reason, the *selection by pattern* rule selects only one of the *2*nd sensor data (*S_c,i+_*_1_) and the *3*rd sensor data (*S_c,i+_*_2_) except the 1st sensor data *(S_c,i_)* in the compression interval.

### 5.5. Selection by Minimum Error Rule

In the SMEP method, given a 2-size compression interval covering and a compression interval for compression, the *selection by minimum error* rule uses two beforehand-selected sensor data from two neighbor compression intervals for sensor data selection. That is, this rule selects the sensor data to minimize error using those two beforehand-selected sensor data. Before the detailed description, we introduce the selection error of a sensor data, for the time being:

**Definition** **16.** The selection error of a sensor data is defined as an error incurred by selecting a sensor data among two or more selectable sensor data.

**Figure 7:**
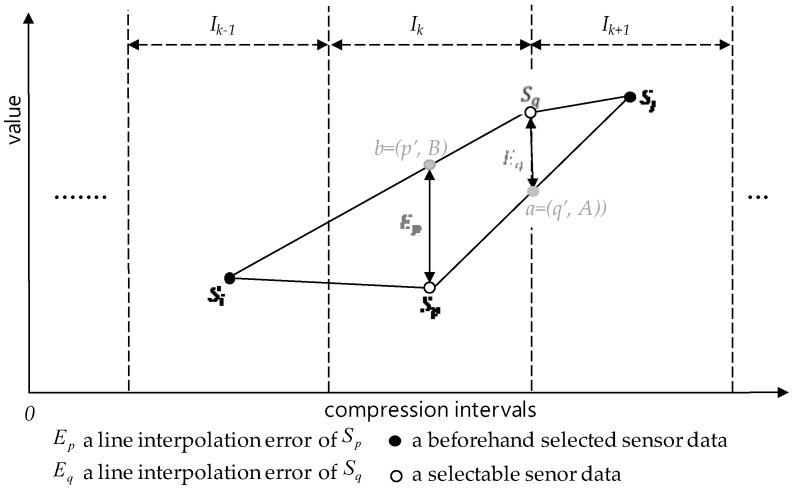
Line interpolation error measure.

Our purpose in compression is to minimize selection errors so that the lost sensor data, i.e., the unselected sensor data, should be restored to sensor data almost similar to the original sensor data. For our purpose, we need an estimation measure to allow compare their selection error sizes each other among sensor data. For this reason, we introduce a line interpolation error measure as one of estimation measures. Figure 7 shows the line interpolation error measure to be used as a selection error measure and it is defined at the below:

**Definition** **17.** *Let be I_k−_*_1_*, I_k_ and I_k+_*_1_ *be compression intervals and let* $S_{i}$ *and* $S_{j}$ *be the beforehand selected sensor data in I_k−1_ and I_k+1_, respectively. For selectable consecutive sensor data, S_p_ and S_q_ (p ≤ q), consider two lines,* $\bar{S_{p}S_{j}}$ *and* $\bar{S_{i}S_{q}}$ *calculated using absolute* positions *of* $S_{i}$ *and* $S_{j}$*. And consider two points, a = (q’, A) and b = (p’, B) on* $\bar{S_{p}S_{j}}$ *and* $\bar{S_{i}S_{q}}$*, respectively, where p’ and q’ are absolute position of p and q. Then,* *line interpolation errors,* $E_{p}$ *and* $E_{q},\;$ *of* $S_{p}$ *and* $S_{q}$ *are defined as follows:*

$$E_{p}=\left|S_{q}-A\;\right|\text{ and }E_{q}=\left|S_{p}-B\;\right|.$$

From now on, we will regard the selection error as the line interpolation error. In the definition, the absolute position for each of all sensor data is used for calculating line interpolation errors. We have a valid reason why we have to use the absolute positions of sensor data in calculating the line interpolation errors: Consider three or more consecutive sensor data to take one or more compressions. They seem likely to have the same distance unit 1 each other at glance. Generally, however, there can be big differences in their absolute positions. For example, consider in Figure 2 three sensor data, *S*_2,0_, *S*_2,1_ and *S*_2,2_, where they are consecutive each other with the same 1 unit distance in the 2nd round compressed sensor data sequence. Their corresponding original sensor data, however, are *S*_0,0_, *S*_0,2_ and *S*_0,7_ and the distance units between them are different with 2 and 5 between *S*_0*,*0_ and *S*_0*,*2_ and between *S*_0*,*2_ and *S*_0*,*7_, respectively.

Exceptionally, the SMEP uses *S_c,_*_0_ for the predetermined sensor data *S_c,i_* in the definition so to select one of *S_c,_*_1_ and *S_c,_*_2_ for *I_c,_*_0_. The reason to do so is because there exists no interval for predetermined sensor data in its left-hand side but if any interval can exit the unique sensor data is the very *S_c,_*_0_ that can be the left boundary sharable sensor data of *I_c,_*_0_.

The SMEP executes the *selection by minimum error* rule for each *I*_2*k*_ and the *selection by pattern* rule for *I*_2*k+*1_ in $C_{S_{c}}$, for *k*=0, 1, …,$\left|C_{S_{c}}\right|$. Therefore, in the definition, the real compression intervals for the SMEP method to apply the *selection by minimum error* rule to the compression are *I*_2*k*_ for *k* = 0, 1,…,$\left|C_{S_{c}}\right|$. Now, focusing on *I*_2*k*_ (*k* = 0, 1,…,$\left|C_{S_{c}}\right|$), we investigate some properties for minimizing the selection error.

**Lemma** **5.** *Let be I_c,_*_2*k−*1_*, I_c,_*_2*k*_ *and I_c,_*_2*k+*2_ *be consecutive compression intervals in* $C_{S^{c}}$ *of S^c^ and let* $S_{c,i}$ *and* $S_{c,j}$ *be the beforehand selected sensor data in I*_2*k−*1_ *and I*_2*k+*2_*, respectively. Given B_p_, an I-bit position sequence of S^c^, for i’, j’, p’ and q’ in the line interpolation error definition,*

*i’* = *(I − 1)2^c^* + *(BitSubstring(B_p_, c, i))*_10_,

*j’* = *(j − 1)2^c^* + *(BitSubstring(B_p_, c, j))*_10_,

*p’* = *(p − 1)2^c^* + *(BitSubstring(B_p_, c, p))*_10_,

*q’* = *(q − 1)2^c^* + *(BitSubstring(B_p_, c, q))*_10*.*_

Moreover, for *A* and *B* in the definition,

$$A=\frac{S_{c,j}-S_{c,4k+1}}{j^{\prime }-p^{\prime }}\left(q\prime -p^{\prime }\right)+S_{c,4k+1}$$

$$B=\frac{S_{c,i}-S_{c,4k+2}}{i^{\prime }-q^{\prime }}\left(p\prime -q^{\prime }\right)+S_{c,4k+2}$$

For the proof of Lemma 5 refer to Appendix L.

**Theorem** **7.** For selectable *S_p_* and *S_q_* in *I*_2*k*_ in the *line interpolation error* definition (Definition 17),

$$E_{p}=E_{4k+1}=\left|\frac{S_{c,j}-S_{c,4k+1}}{j^{\prime }-p^{\prime }}\left(q^{\prime }-p^{\prime }\right)-(S_{c,4k+2}-S_{c,4k+1})\right|$$

and

$$E_{q}=E_{4k+2}\;=\left|\frac{S_{c,i}-S_{c,4k+2}}{i^{\prime }-q^{\prime }}\left(q\prime -p^{\prime }\right)-\;(S_{c,4k+2}-S_{c,4k+1})\right|$$

For the proof of Theorem 7 refer to Appendix E.

Until now, in this section we have used the terminology *selection by minimum error* rule without its exact formal definition. Now, the definition of this rule is formally defined as the below:

**Definition** **18.** *Given the two selectable sensor data, S_c,_*_4*k+*1_ *and S_c,_*_4*k+*2_*, in I_c,_*_2*k*_ *in* $C_{S_{c}}$ *of S^c^_,_ the selection by minimum error rule is defined as a rule to select S_c,i_ as S_c+_*_1*,k*_ *in the c+1 th round compression such that:*

$$p=\underset{i\in \left\{4k+1,\;4k+2\right\}}{arg\;min}\left(E_{i}\right)$$

## 6. Main SMEP Elements

SMEP algorithms create and use a few of data structures as main elements for the compression of sensor data sequences. These data structures are *DataQueue*, *Zones*, ZoneBpSeq, *ZoneCompHistory*, and *AuxDataQueue*. Moreover, each information in these main elements is transmitted to one or more consumer (i.e., destination) sites in order that they decompress and recover compressed and lost sensor data using this information. This section presents each of these data structures, their operations, and the related properties significant to the SMEP algorithms.

### 6.1. Data Queue and Zones

*DataQueue* is an array that saves a ground sensor data and compressed sensor data and the compression is executed on this *DataQueue*. Each compression makes the number of the targeted ground or compressed sensor data in *DataQueue* reduce to the half. For this reason, the size of *DataQueue* must be *2^m^ + 1*. Furthermore, *Zones* array information corresponding to *DataQueue* ranges is a basis on each compression. *Zones* data structure is defined as the below:

**Definition** **19.** *Given a data queue, DataQueue[0..2m], with the size 2^m^ + 1, a Zones with respect to the data queue is defined as a data structure with the size m, this is, Zones[0..m − 1], as follow:*

*(1)*  *Zones[0]* *corresponds to the range DataQueue[k] for k, 0 ≤ k ≤ 2^m−1^.*
*(2)*  *Zones[i] for i = 1,…,m − 2 corresponds to the range DataQueue[k] for k*, $\sum_{j=0}^{j=i-1}2^{m-j-1}+1\leq k\leq \sum_{j=0}^{j=i-1}2^{m-j-1}+2^{m-i-1}$.
*(3)*  Zones[m − 1] corresponds to the range DataQueue[k] for k, 2^m^ − 1 ≤ k ≤ 2^m^.
*(4)*  Each value of Zones[m − 1] represents the number of compressions through which all sensor data in the data queue corresponding to the Zones[m − 1] have been compressed.

**Figure 8:**
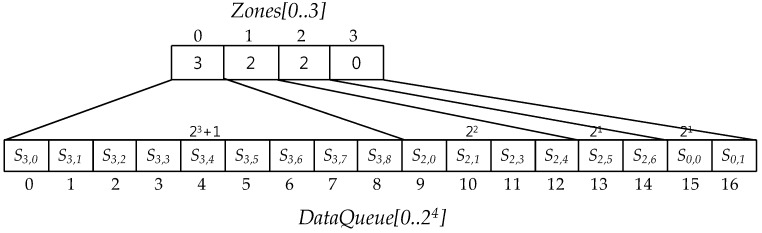
*Zones* example with respect to *DataQueue[0..2^4^]*.

When a zone corresponds to a range of DataQueue, we call *the zone covers the range*. Figure 8 illustrates *Zones[0..3]* with respect to *DataQueue[0..2^4^]*. In this figure *Zones[0]* covers a range from *S*_3*.*0_ to *S*_3,8_. This range is relevant to one plus a half of *DataQueue* size as an area to save the selected sensor data when *DataQueue* is full of ground or compressed sensor data. In Figure 8, all sensor data in this range have been selected through three compressions. *Zones[1]* covers a range from *S*_2.0_ (*DataQueue[9]*) to *S*_2*,*4_ (*DataQueue[12]*) taken through two compressions and the number of sensor data in this range is 2^2^. *Zones[2]* and *Zones[3]* cover a range between *S*_2.5_ (*DataQueue[13]*) and *S*_2,6_ (*DataQueue[14]*) and between a range of *S*_0.0_ (*DataQueue[15]*) and *S*_0,1_ (*DataQueue[16]*), respectively. In particular, the range that *Zones[3]* covers does not path through any compression in Figure 8.

Now, we introduce operations on the *Zones* data structure.

**Definition** **20.** *The operations on* $Zones\left[0..m-1\right]=[c_{0},\;c_{1},\ldots ,\;c_{m-1}]$ *are defined as follows:*

Rule 1. *At first, ground sensor data are saved into DataQueue starting from DataQueue[0], this is, the first location of the DataQueue region corresponding to Zones[0], and the values of Zones are* $\overset{m}{\overset{︷}{\left[0,\;0,\ldots \ldots \ldots ,0\right]}}$ *until DataQueue is filled up with ground sensor data and.*
Rule 2. *Given* $Zones=[c_{0},\;c_{1},\ldots ,\;c_{m-2},c_{m-1}]$, if $c_{m-1}=c_{m-2}$ *after a compression at the last zone Zones[m − 1]*, *then*
  (i) *the* $c_{m-2}$*th compression on DataQueue is executed on the zones from* $c_{k}$ *such that* $k=min\left\{i|c_{i}=c_{m-1}\;\right\}$ *to* $c_{m-1}$,
  (ii) *the result of Zones after the i) compression is* $Zones=[c_{0},\;c_{1},\ldots ,\;c_{k}+1,\overset{m-k-1}{\overset{︷}{0,\ldots ,0}}]$.
  (iii) *Additionally, new ground sensor data are saved into DataQueue starting from DataQueue[*$\sum_{j=0}^{j=k}2^{m-j-1}+1]$, *this is, the first location of DataQueue covered by* $Zones\left[c_{k+1}\right]$.

**Figure 9:**
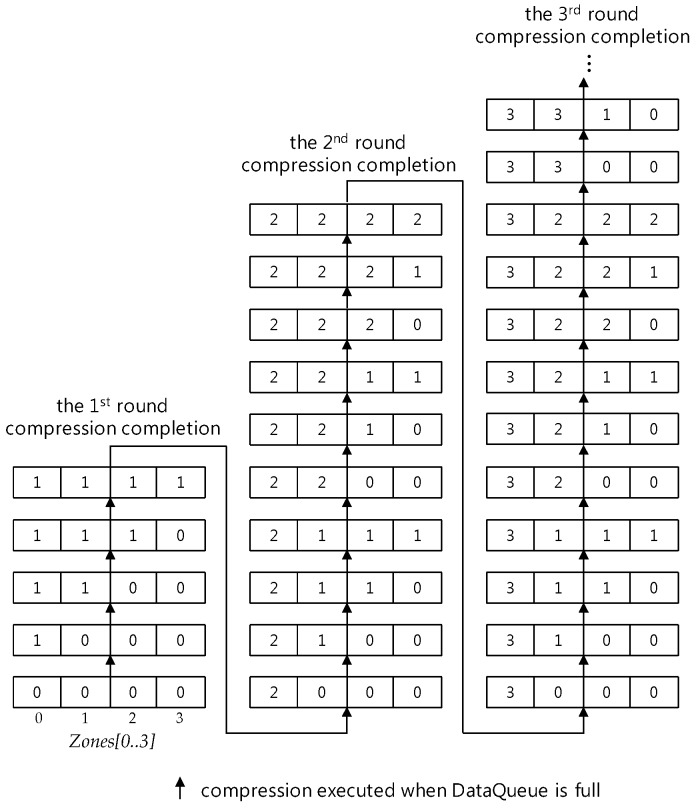
Changes of *Zones* values in compressions and round compressions.

Figure 9 shows the changes of *Zones* values in compressions and round compressions according to the *Zones operation rules*. The arrow in the figure represent the compression executed when *DataQueue* is full. Note the round compression consists of one or more compressions and the round compression is finished when all values in *Zones* are same. At the beginning of the *SDSC* mode *Zones* values are 0,0,0,0 according to *Zones operation Rule 1* and they are also the same values until *DataQueue* is full firstly. As soon as *DataQueue* is filled up, the first compression proceeds for whole sensor data in it, the first compressed sensor data are saved to the range of *DataQueue* covered by *Zone[0]* and the value of *Zones[0]* is 1, which means the *DataQueue* sensor data in *Zones[0]* have been compressed once. The number of compressed sensor data is the same as the size of the range covered by *Zones[0]*. After the compression, *Zones[1]*, *Zones[2]* and *Zones[3]* values become all 0, which means sensor data in these zone ranges don’t take any compression so to be able to save the new generated sensor data and to compress them. When ranges covered by these 0 value zones are full again, the second compression makes the second zone *Zones[1]* value be 1, the number of compressions, and it makes Zones*[2]* and Zone*[3]* values be all zero. When values of *Zones[0..3]* are all 1s, the 1st round compression is completed and the 2nd round compression begins. Generally, if all values of *Zones[0..m − 1]* are same with *c* in the *c*-th round compression then the *c*-th round compression is completed and the *c + 1* th round compression begins. In this case, the value of *Zones[0]* is one up so to be *c + 1*, the other *Zones* values are all 0s, and another new compressions begin whenever DataQueue space covered by 0 value zones are full. These compression processes continues repeatedly until all *Zones* values are same with *c + 1*.

### 6.2. Zone Bit Sequence

Now, we introduce *ZoneBpSeq[0..m − 1]* that is an array of references to *I-bit position* sequences of *Zones*. Numbers of compressions in zones of *DataQueue* generally differ from each other and the length of I-bit position of sensor data in each zone can differ from other zone lengths, too. Therefore, we cannot handle all the sensor data in *DataQueue* as a sensor data sequence in compressions. Consequently, we have no way but to treat *DataQueue* sensor data with a sensor data sequence per each zone. I-bit position lengths of sensor data in the same zone are all the same since they have passed through the same number of compressions. For this reason, we use *ZoneBpSeq[0..m − 1]* as an array of pointers to refer to I-bit position sequences of the sensor data sequences corresponding to each zones of *DataQueue*.

**Definition** **21.** *Given a zone of DataQueue, its zone bit sequence is defined as the I-bit position sequence corresponding to a sensor data sequence in the zone and we call ZoneBpSeq[0..m − 1]* *a zone bit sequence array.*

With the arrays *Zones* and *ZoneBpSeq* we can seek the absolute position of any sensor data in *DataQueue* according to the following theorem (For the proof refer to Appendix F).

**Theorem** **8.** *Let P_i_ be the absolute position for the sensor data DataQueue[i] for any i such that 1 ≤ I ≤ 2^m^. Given a Zones[0..m − 1]* *and ZoneBpSeq[0..m − 1],*

*(i)*  *i exits in the zone* $k=max\left\{\;t\;|\;0<\left(i-2^{m-t}\cdot \left(2^{t}-1\right)\right)\;\right\}$ for 1 ≤ *I* ≤ 2*^m^ −* 1 and *i exists in the zone m − 1 for i =* 2*^m^*.
*(ii)*  $P_{i}=\{\;\begin{array}{c} \;0\;\mathrm{for}\;i=0\;\\ \sum_{j=0}^{j=k-1}2^{m-j-1}\cdot 2^{Zones\left[j\right]\;}+\;(i-\sum_{j=0}^{j=k-1}2^{m-j-1}-1)\cdot 2^{Zones\left[k\right]}+\;\mathrm{for}\;i\neq 0\;\mathrm{and}\;k\geq 0\;\\ {\left(BitSubstring(ZoneBpSeq\left[k\right],\;Zones\left[k\right],\;i-\sum_{j=0}^{j=k-1}2^{m-j-1})\right)}_{10}+1\; \end{array}$

### 6.3. Zone Compression History

**Figure 10:**
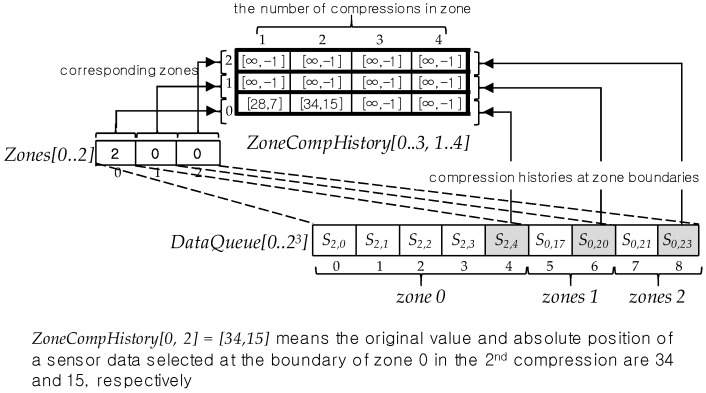
Historical sensor data in compressions.

In every compression, the sensor data *S*_c,0_ in the zone 0 is selected unconditionally in the compression interval sequence in the zone. In fact, *S*_c,0_ in the zone 0 plays a role as a base in sensor data selection by the first application of *selection by minimum error* rule in the zone *0*. We call such a sensor data like *S_c,_*_0_ the *base sensor data* in the zone of *DataQueue*. In other words, a base sensor data is the first sensor data positioned in the first interval in a zone of *DataQueue* and it has a role as a base in applying the first *selection by minimum error* rule in the zone. By the way, a problem is that there is no base sensor data in all zones except the zone 0 so to prevent from selecting a new sensor data from the first interval in the first zone for the compression. To resolve this problem, we make the last selected sensor data in the previous zone be a base for the compression of a sensor data sequence after that zone. In spite of doing that, another problem occurs if there is among zones no history about sensor data selected in every compression. Figure 10 shows such an example. In this figure, the sensor data sequence between the zone 1 and the zone 2 should be compressed and the numbers of compressions in the zone 0 and in the zone 1 are different as 2 and 0, respectively. In this case, however, there is no way for compression without the last selected sensor and absolute position in the zone 0 as a historical sensor data in the 1st compression. Because there is no base sensor data for the 1 th compression. For this reason, we introduce *ZoneCompHistory*, an array, as the very data structure to keep the historical information about zone compressions. As shown in Figure 10, the rows in the *ZoneCompHistory* array corresponds to zones of *DataQueue* and its columns correspond to the number of compressions. Moreover, its value is a pair of the value and absolute position of the sensor data selected in the zone through compression.

As an example, in Figure 10, *ZoneCompHistory[0,1]* tells the sensor data with 28 as its value and 7 as its absolute position had been selected as a last sensor data in the zone 0 through the 1 st compression. This sensor data is the very base for the 1st compression of a sensor data sequence between zones 1 and 2.

### 6.4. Auxiliary Data Queue

**Figure 11:**
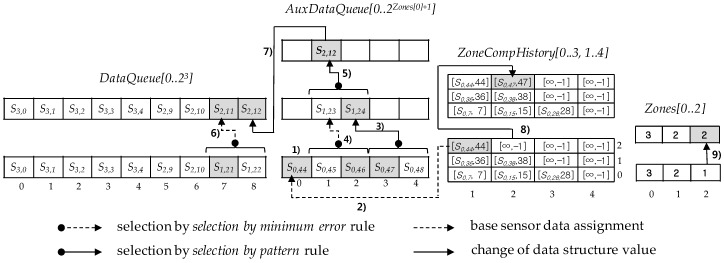
Compression process on last zone.

As long as the number of compressions in the last zone of *DataQueue* is less than the number of compressions in its previous zone, there occurs no new compression with its neighbor zones even though *DataQueu*e is filled up with compressed sensor data. In this situation, *AuxDataQueue*, an auxiliary data queue, is used for new ground sensor data to be inserted into and it is also used to adjust the number of compressions in the last zone until that number is to be equal to the number of compressions in its previous zone. Using *AuxDataQueue*, the number of the last zone compressions increases one by one until it equals to the number of its previous zone compressions. Figure 11 shows such an example to use an auxiliary queue for a compression on the last zone. Figure 11 also illustrates in turn the main steps and changes of the last zone compression process with numbering.

Given *Zones[m − 1]*, the number of compressions in the last zone, *AuxDataQueue[0..2*^*Zones[m−*1*]+*1^*],* an array with the size *2^zones[m−^*^1*]+*1^ *+1*, is required to increase the number of the last zone compressions up one more (^1)^ in Figure 11). Note that in Figure 11 *m*, *Zones[m − 1]*, and *AuxDataQueue[0,...,2^Zones[m−1]+1^]* are 3, 1, and *AuxDataQueue[0..4]*, respectively. Specially*, AuxDataQueue[0]* is used as the base sensor data assigned from *ZoneCompHistory[m −* *1, c]*s for *c* between 1 and *Zones[m − 1]* (^2)^ in Figure 11). A new compression begins when *AuxDataQueue* becomes full and continues until only one selected sensor data remains in the queue through *Zones[m −* *1]* + 1 compressions. Of course, the rules of *selection by pattern* and *selection by minimum error* are applied alternately to auxiliary queue compressions (^3), 4), 5)^ in Figure 11). Moreover, the final compression on auxiliary queue is always complete with the *selection by pattern* rule (^5)^ in Figure 11). Next, one of two sensor data in the last zone is selected according to the *selection by minimum error* rule, using *ZoneCompHistory[m −* *2, Zones[m −* *1] + 1]* and the remaining auxiliary queue sensor data as a base and a sensor data selected through the *selection by pattern* rule, respectively (^6)^ in Figure 11). This selected sensor data and the remaining auxiliary queue sensor data are replaced as the values before and at the last position in *DataQueue*, respectively (^7)^ in Figure 11). During these steps, *I-bit* positions of each selected sensor data are calculated according to *I-bit position* definition (Definition 11) and they are reflected to find their absolute positions and to make an *I-bit* position sequence of the last zone. Accordingly, *ZoneCompHistory* is modified with the last selected sensor data value and its absolute position (^8)^ in Figure 11). Finally, the last value of *Zones* is also changed to the same value as the *Zones* value corresponding to the zone before the last zone when the compression on the two last sensor data has been finished (^9)^ in Figure 11).

## 7. SMEP Algorithms

Compression algorithms to execute the SMEP method use the rules, data structures, operations, and theorems in Section 5 and Section 6. For compressions, these algorithms keep and follow *Zones operations definition Rules* in Definition 20 partly or overall. The *AuxDataQueueCompression* algorithm introduced in Section 7.1 as a smallest procedure unit executes compressions on a ground sensor data sequence in *AuxDataQuque*. The *LastZoneCompression* algorithm introduced in Section 7.2 compresses two sensor data in the last zone using *AuxDataQueue Compression*. *EquivalentZonesCompression* algorithm introduced in Section 7.3 performs compression over consecutive zones with the same number of compressions according to *Zones operations definition rule* when *DataQueue* is full and the number of compressions of the last zone is same as that of its neighbor zone. Following *overall Zones operations definition rules* the SMEP main algorithm introduced in Section 7.4 controls and manages compressions in various cases using the above algorithms. Now, we introduce these algorithms in turn in this subsections.

### 7.1. AuxDataQueue Compression Algorithm

The *AuxDataQueueCompression* algorithm is the algorithm for compressing a sensor data sequence in *AuxDataQueue.* This algorithm is illustrated Algorithm 1.

**Algorithm 1:**
*AuxDataQueueCompression(AuxDataQueue, m)*Parameters *m*: the number of zones such that *DataQueue[0..2^m^]* Global Variables Local Variables 01 *zoneCValue*: the number of compressions of sensor data sequence in the *DataQueue[m − 1]* zone 02 *c*: the number of compressions for sensor data sequence in *AuxDataQueue* 03 *DQMaxABP*: the greatest absolute position covered by *DataQueue* 04 *ZoneBpSeq0, ZoneBpSeq1*: bit string variables for an *I-bit* sequence generation Procedure 01 Set *zoneCValue = Zones[m − 1];* 02 Set *DQABP = *$\sum_{j=0}^{j=m-2}2^{m-j-1}\cdot 2^{Zones\left[j\right]\;}+2\cdot 2^{Zones\left[m-1\right]}$ 03 Initialize *c = 1* 04 Initialize *ZoneBpSeq0 =*
ϵb (an empty bit string) 05 while (*c ≤ czoneCValue*) do 06 Set *C* to the 2-size compression interval covering on a sensor data sequence in     *AuxDataQueue[0..2^zoneCValue+*1*^]* 07 Initialize *ZoneBpSeq1 =*
ϵb (an empty bit string) 08 Set *AuxDataQueue[0] = ZoneCompHistory[m − 1, c].Value* 09 for each $I_{c-1,2k}$
*in C such that 0 ≤ k ≤ 2^zoneCValue^) do * 10   Select *S_c_*_−1,*j*_ from $I_{c-1,2k+1}$ by the *selection by pattern* Rule 11   Select *S_c_*_−1,*p*_ from $I_{c-1,2k+2}$ by the *selection by minimum error* Rule using         *ZoneCompHistory[m − 1, c]* as a base sensor data and its absolute position,            this is, $p=\underset{i\in \left\{4k+1,\;4k+2\right\}}{arg\;min}\left(E_{i}\right)$ 12   Set *DataQueue[k + 1] = S_c−1,p_* 13   Set *DataQueue[k + 2] = S_c−1,j_* 14   Set *ZoneBpSeq1* = *ZoneBpSeq1 Θ I-bit position(S_c,k_*_+1_*) Θ I-bit position(S_c,k_*_+2_*)*
       by using *ZoneBpSeq0* 15 endfor 16 Set *ZoneBpSeq0* = *ZoneBpSeq1* 17 Set *c* = *c + 1* 18 endwhile 19 Select *S_c,j_* from $I_{c,0}$ by the *selection by pattern* rule 20 Set *AuxDataQueue[1]* = *S_c,j_* 21 Set *ZoneBpSeq0* = *I* − *bit position(S_c+1,1_)* by using *ZoneBpSeq1* 22 Set *j* = the position of *S_c,j_* with respect to *AuxDataQueue* by using ZoneBpSeq0 23 return *[AuxDataQueue[1]*, *DQMaxABP+j*, *ZoneBpSeq0]* Endprocedure

This algorithm increases one more the number of compressions in the last zone of *DataQueue*. The local variable *zoneCValue* is used as the number of compressions in the last zone. The *zoneCValue-time* compression part over *AuxDataQueue* appears from the procedure line 5 to the line 18. Here, note that the *selection by pattern rule* and the *selection by minimum error* rule are applied alternately in lines 10 and 11. The lines 14, 16, 20, and 21 generates ultimately the I-bit position of the finally selected sensor data with respect to a ground sensor data sequence in *AuxDataQueue* before *AuxDataQueueCompression* algorithm execution. With this position and the *DQMaxABP* variable in the procedure line 2, the absolute position of the sensor data finally compressed through this algorithm can be obtained as *DQMaxABP+j*, one of return values in the line 23, where the *DQMaxABP* variable value is calculated in the line 2 as the greatest absolute position covered by the *DataQueue*. This position is very important in that it is recorded into *ZoneCompHistory* with the selected sensor data value and it can be used as the absolute position of a base sensor data in the future compressions. Finally, the algorithm executes the *zoneCValue th compression in the line 19 applying the selection by pattern* rule, selects only one sensor data, and returns its value, absolute position and I-bit position.

### 7.2. Last Zone Compression Algorithm

The *LastZoneCompression* is a procedure that reads ground sensor data and compresses them using *AuxDataQueueCompession* procedure so to increase one more the number of compressions of the last zone. The *LastZoneCompression* procedure is called by the *SMEP* main procedure introduced in Section 7.4 when the last zone becomes full and the number of compressions in the last zone is less than the number in its previous neighbor zone. The last zone compression algorithm is shown in Algorithm 2.

**Algorithm 2:**
*LastZoneCompression(DataQueue, m)*Parameters01 *m*: the number of zones such that *DataQueue[0..2^m^]*Global Variables01 *AuxDataQueue[0..2^Zones[m−*1*]^^+^*^1^*]*: an auxiliary data queue for compressing a sensor sequence in the last zone02 *Ap*: a variable for the absolution position of the latest ground sensor dataLocal Variables01 *c*: the number of compressions for a sensor data sequence in *Zones[m − 1]*02 *AuxDQinx: an index for AuxDataQueue*03 *ZoneBpSeq0*: a temporal bit string variable for I-bit sequenceProcedure01 Set *c=Zones[m − 1]*02 Initialize *ZoneBpSeq0 =*
ϵb (an empty bit string)04 Allocate memory *AuxDataQueue[0..2^c+*1*^]*05 Initialize *AuxDQinx* = *1*06 while(*AuxDQinx ≤* 2^c+1^) do07 Set *Ap=Ap+1*08 Read *a ground sensor data S*_0*,Ap*_ from a sensor09 Insert *S*_0*,Ap*_ into *AuxDataQueue[AuxDQinx]*10 if (*receive(CRmessage) =* true)11 then return *‘FinishMode’*12 else Set *AuxDQinx=AuxDQinx+1*13 Continue14 endif15 endwhile16 Set *[Value, j, ZoneBpSeq0] = AuxDataQueueCompression(AuxDataQueue, m)*17 Select *S_c,p_* from the $2^{m-1}-1$ positioned interval of *DataQueue* by the *selection by minimum*   *error* rule by using *ZoneCompHistory[m − 2, c + 1]* as a base sensor data and its absolute   position and by using *[Value, j]* as the value and absolute position of the sensor   data selected in the next interval in the c+1 th compression,      this is, Set $p=\underset{i\in \left\{2^{m}-1,\;2^{m}\right\}}{arg\;min}\left(E_{i}\right)$18 Set *DataQueue[2^m^* − 1*] = S_c,p_*19 Set *ZoneBpSeq0 = I-bit position(DataQueue[*2*^m^* − 1*]) Θ ZoneBpSeq0* by using *ZoneBpSeq[m − 1]*20 Set *DataQueue[*2*^m^]* = *Value*21 Set *ZoneBpSeq[m − 1] = ZoneBpSeq0*22 Set *ZoneCompHistory[m − 1, c+1] = [Value, j]*23 return *‘Continue’*Endprocedure

The *LastZoneCompression* procedure allocates the *AuxDataQueue* array space in order to make the last zone be taken to the one more compression (procedure line 4). Hence, the size of *AuxDataQueue* array space must be 2*^Zones[m−1]+1^*. Next, this procedure begins to read ground sensor data and inserts them into *AuxDataQueue* (from procedure line 6 to line 15). Whenever the procedure reads a ground sensor data, it checks if the communication failure has been recovered using *Receive(CRmessage)* function (procedure line 10). If the check is true, the procedure returns to the calling procedure, the SMEP main procedure, and informs the recovery from the communication failure to that procedure by returning *‘FinishMode’* (procedure line 11). After filling up *AuxDataQueue* the procedure calls the *AuxDataQueueCompression* procedure to compress a ground sensor data in *AuxDataQueue* (procedure line 16). The *AuxDataQueueCompression* procedure returns a finally selected sensor data value, its absolute position, and its zone bit sequence (procedure line 16). The procedure uses the returned value and absolute position to select one between two last sensor data by the *selection by minimum error* rule (procedure line 17). The finally selected sensor data and the returned value are inserted into *DataQueue[2m − 2]* and *DataQueue[2m − 1]*, respectively, as the results of the last zone compression (procedure line 18 and line 20). The I-bit position of the finally selected sensor data and the returned bit sequence are used for a last zone bit sequence (procedure line 19 and line 21). Then, the new last sensor data value and its absolute position in the last zone are newly recorded into *ZoneCompHistory[m − 1,c+1]* for the next compressions (procedure line 22), where c is the number of the last compression in the last zone before calling this procedure.

### 7.3. Consecutive Equivalent Zones Compression Algorithm

Let us say that the zones are *equivalent* when zones are same in numbers of compressions. *EquivalentZonesCompression* is a procedure that compresses the consecutive zones equivalent with the last zone, and that keeps *Rule 2 (i)* and (*ii)* in the *Zones operations* definition (Definition 20). This procedure also returns to the *SMEP* main procedure the value and absolute position of the last selected sensor data, where this sensor data is the last of the beginning zone among consecutive equivalent zones after compressing their sensor data. The *SMEP* main procedure calls this procedure to pass the beginning zone among consecutive equivalent zones to the parameter *StartZone*, as shown in the Algorithm 3.

*DQGP* is calculated as the greatest position (index) among *DataQueue* positions (indices) covered by zones before the *StartZone* zone. This position is used to calculate *DataQueue* positions of sensor data in *DataQueue* from the *StartZone* zone to the last *m − 1* zone (procedure line 2). In fact, a sensor data sequence from the *StartZone* zone to the last zone is the same as the sensor data sequence from *DataQueue[DQGP+1]* to *DataQueue[*2*^m^]*. At this time, if *StartZone* is not zero, the *ZoneCompHistory[StartZone, c].Value* is used as a base sensor data and *ZoneCompHistory[StartZone, c]. Position* is used as its absolute position (procedure line 4). The compression proceeds over the 2-size compression interval covering on this sensor data sequence (from procedure line 4 to line 11). In this compression, the procedure applies alternately the *selection by pattern* rule and the *selection by minimum error* rule for the odd number intervals and the even number intervals, respectively (procedure line 6 and line 7). Here, *DQGP* is used to calculate the *DataQueue* position to save the sensor data selected for each interval (procedure line 8 and line 9). The sensor data sequence from the *StartZone* zone to the last *m − 1* zone is compressed to the *Startzone* zone, where this is proved in the last of this section. Additionally, the zone bit sequence about the *StartZone* zone is generated during compression using I-bit positions of *S_c,p_* and *S_c,j_* and the zone bit strings corresponding to them (procedure line 10 and line 12). The procedure calculates the absolute position of the last selected sensor data in the compression using *Zones[StartZone]* and *ZoneBpSeq[StartZone]* according to the Theorem 8 (procedure line 13), and returns this last sensor data value and absolute position which are to be saved to *ZoneCompHistory[StartZone, Zones[StartZone] + 1]* by the *SMEP* main procedure.

Finally, we prove the below theorem (For the proof refer to Appendix G):

**Theorem** **9.** In the compression for the sensor data sequence in consecutive equivalent zones from the StartZone zone to the last m − 1 zone in DataQueue, this sensor data sequence is compressed to the Startzone zone.

**Algorithm 3:**
*EquivalentZonesCompression(DataQueue, StartZone, m)*Parameters01 *StartZone*: a compression start zone02 *m*: the number of zones such that *DataQueue[0..2^m^]*03 *c*: the number of compressions for a sensor data sequence in the zone *StartZone*Global VariablesLocal Variables01 *DQGP*: the greatest position (index) among *DataQueue* positions (indices) covered by zones before the zone *StartZone*. This position is used to calculate *DataQueue* positions from the *StartZone* zone to the last *m − 1* zone02 *APos*: an absolute position of the last sensor data in the zone *StartZone* after the compressionProcedure01 Set *c = Zones[StartZone]*02 if *DQGP =0* then *Set DQGP = 0 *else Set *DQGP =*
$\sum_{j=0}^{j=StartZone-1}2^{m-j-1}$ endif03 Initialize *ZoneBpSeq0 = ϵ b* (an empty bit string)04 Set $C_{DQGP}$ = a 2-size compression interval covering with respect to a sensor data sequence from *DataQueue[DQGP+1]* to *DataQueue[2^m^]* (if *StartZone*≠0, set $C_{DQGP}$ including to this sensor data sequence *ZoneCompHistory[StartZone − 1, c + 1].Value* as a base sensor data and its absolute position as *ZoneCompHistory[StartZone − 1, c + 1].Position*)05 for each $I_{c,2k}$
*in*
$C_{DQGP}$ such that *k* is an integer and *0*
*≤*
*k <*
*2^m−StartZone−^*^2^ do06 Select *S_c,j_* from $I_{c,2k+1}$ by the *selection by pattern* rule07 Select *S_c,p_* from $I_{c,2k}$ the *selection by minimum error* Rule,      this is, $p=\underset{i\in \left\{4k+1,\;4k+2\right\}}{arg\;min}\left(E_{i}\right)$08 Set *DataQueue[DQGP+2k+1] = S_c,p_*09 Set *DataQueue[DQGP+2k+2] = S_c,j_*10 Set *ZoneBpSeq0 = ZoneBpSeq0 Θ I-bit position(S_c+_*_1*, DQGP+*2*k+*1_*) Θ I-bit position(S_c+_*_1*, DQGP+*2*k+*2_*)* using    *I-bit position(S_c,p_)*, *I-bit position(S_c,j_)* and the zone bit strings corresponding to *p* and *j*11 endfor12 Set *ZoneBpSeq[StartZone] = ZoneBpSeq0*13 Set *APos* = an absolute position of *S_c,j_* calculated by the theorem using *Zones[StartZone]*   and *ZoneBpSeq[StartZone]*14 return *[S_c,j_, APos]*Endprocedure

### 7.4. Main Algorithm

The SMEP main procedure proceeds to save and compress the growing ground sensor data sequence controlling *Zones operations Rule 1* and *Rule 2* in the Definition 20 in Section 6.1. This procedure uses the data structures and procedures already introduced in previous sections. This algorithm is illustrated in Algorithm 4.

At first, the produce initializes *Zones* and *ZoneCompHistory* data structures, the absolute position variable *Ap*, the *DataQueue* index *DQinx* and the zone index variable *ZNinx* (from procedure line 1 to line 7). Then, the procedure reads and inserts a ground sensor data into *DataQueue* (procedure line 9 and line 10).

If *DataQueue* is not full but the communication failure is not still recovered (procedure line 12), then the procedure continues to read and insert a new sensor data into *DataQueue* (procedure line 14 and line 15). If the communication failure is recovered (procedure line 12) then the procedure returns all sensor data in *DataQueue*, the *Zones* data, the *ZonesBpSeq* data*,* the *ZoneCompHistory* data and the absolute position *Ap* of the last ground sensor data to *CDTR* mode via *CRP* mode (procedure line 13). The *CDTR* mode procedure will transmit them to the monitoring and control center (i.e., destination site), in which the original ground data and the lost sensor data are restored, ultimately. How to transmit in the *CDTR* mode via the *CRP* mode is beyond our issues.

If *DataQueue* becomes full, then the procedure continues to compress the last zone while the number of the compressions of the last zone is less than that of its previous zone (from procedure line 18 to line 24). For the last zone compression the procedure calls the *LastZoneCompression* procedure. As described before, the *LastZoneCompression* procedure returns *‘FinishMode’* when it has received *CR* message in the middle of reading and inserting a new ground sensor data (procedure line 10 and line 11 in Algorithm 4). At this time the procedure returns *DataQueue, Zones, ZonesBpSeq, ZoneCompHistory, Ap*, and *AuxDataQueue* essential for the original and lost sensor data to be recovered (procedure line 20, line 21, and line 22). If the procedure doesn’t receive any *CR* message, it increases one more the number of compressions of the last zone since (procedure line 23).

When the last zone and its previous zone become equivalent, according to *Zones operations rule,* the procedure finds the start zone of the consecutive equivalent zones (procedure line 25).

**Algorithm 4:**
*Main SMEP (Selection by Minimum Error and Pattern)*Constant01 *cmax*: the maximum number of compressionsGlobal Variables01 *DataQueue[0..2^m^]: a data queue array for saving sensor data or compressing a sensor data sequence*02 *Zones[0..m − 1]: an array of zones with respect to DataQueue[0..2^m^], where Zone[i] ≤ cmax*03 *AuxDataQueue[0.. 2^Zones[m−*1*]^]:* an auxiliary data queue for the compressing a sensor sequence   in the last *m − 1* zone04 *ZoneBpSeq[0..m − 1]: an array of pointers to refer to I-bit position sequences of the sensor data sequences*   *corresponding to each zones*05 *ZoneCompHistory[0..m − 1, 1..cmax]: an array of pairs, [S, p]s, where ZoneCompHistory[i, c] = [S, p]*   *and S and p are the sensor data value and its absolute position of the last c-th compressed*   *sensor data in the zone i*06 *Ap*: a variable for the absolution position of the latest ground sensor dataLocal Variables01 *DQinx*: an index variable for the latest sensor data insertion to *DataQueue*02 *ZNinx: an index variable in Zones array*Procedure01 for each *k such that 0*
*≤ k*
*≤*
*m − 1* do02 Initialize *Zones[k] = 0*03 for each *l* such that *0*
*≤ l*
*≤*
*cmax* do04 Initialize *ZoneCompHistory[k, l] = [∞, −1]*05 endfor06 endfor07 Initialize *Ap = 0, DQinx = 0, and ZNinx=0*08 while(true) do09 Read *a ground sensor data S*_0*,Ap*_ from a sensor10 Insert *S*_0*,Ap*_ into *DataQueue[DQinx];*11 if (*DQinx ≤ 2^m^*)12 cthen if (*receive(CRmessage) =* true)13  then return [*DataQueue, Zones, ZonesBpSeq, ZoneCompHistory, Ap*]14  else Set *Ap=Ap+1, DQinx=DQinx+1*15      Continue16  endif17 else Set *ZNinx*=*m − 1*18  while(*Zones[ZNinx−1] < Zones[ZNinx]*) do19      Set *RFsate*=*LastZoneCompression(DataQueue, m)*20      if(*RFstate=‘FinishMode’*)21      then return [*DataQueue, Zones, ZonesBpSeq, ZonesCompHistory, Ap, AuxDataQueue*]22      endif23      Set *Zones[ZNinx]=Zones[ZNinx]+1*24  endwhile25  Find Start=min{ k | Zones[k]=Zones[ZNinx] }26  Set *ZoneCompHistory[Start, Zones[Start]] = EquivalentZonesCompression(DataQueue, StartZone, m)*27  Set *Zones[Start]=Zones[Start]+1*28  for each *k* such that *Start + 1*
*≤ k*
*≤*
*m − 1* do29    Set *Zones[k]=0*30      Set *ZoneBpSeq[k]=*null31    for each *l* such that *0*
*≤ l*
*≤*
*Zone[k]* do32  Initialize *ZoneCompHistory[k, l] = [∞, −1]*33    endfor34  endfor35  Set *DQinx =*
$\sum_{j=0}^{j=Start}2^{m-j-1}+1$36 endif37 endwhileEndprocedure

Then, the procedure calls the *EquivalentZonesCompression* procedure to compress these zones into the start zone and it saves the return values, which are the last selected sensor data and its absolute position, to *ZoneCompHistory* for the future compression preparation as the base sensor data and its absolute position of the next zone (procedure line 26). The procedure also increases one more the number of compressions of the last zone (procedure line 27) because the sensor data sequence in equivalent consecutive zones has been one more compressed into the start zone. As the sensor data sequence from the start zone to the last zone shrinks to only the start zone through the consecutive equivalent zones compression, the *DataQueue* memory spaces corresponding to the remaining zones are empty and the first position of the immediate zone of the start zone is the beginning *DataQueue* location to save the new ground sensor data. Thus, the procedure must initialize the data structure parts corresponding to these empty zones, such as the values from the immediate zone to the last zone in *Zones*, *ZoneBpSeq*, and *ZoneCompHistory*. The lines from procedure line 28 to line 34 reflect these initializations. The first position to save a new sensor data is calculated to the *DQinx* variable in procedure line 35.

## 8. Compressed Sensor Data Decompression and Lost Sensor Data Recovery

The final consumers of the return values from *SMEP* main are one or more destinations that receive the values via a wireless sensor network and one or more existing networks, and use them with their own purpose. One of main consumers is generally a monitoring and control center that monitors external or internal environments of each sensor nodes using sensor data transmitted by them, and controls them by sending control messages to sensor nodes if necessary. In order for the final consumer to use the *SMEP* main return values, the consumer must decompress them and recover the unselected sensor data during saving and compressing sensor data. In this section, we introduce algorithms for the decomposition of compressed sensor data and the recovery of lost sensor data.

### 8.1. Sensor Data Line Interpolation Algorithm

Sensor Data Line Interpolation Algorithm uses the line interpolation method to recover the lost sensor data. The line interpolation is a very simple method to interpolate the unknown values of points between two known endpoints. Algorithm 5 illustrates *SensorDataLineInterplolation*, a line interpolation algorithm for lost sensor data recovery:

**Algorithm 5:**
*SensorDataLineInterplolation(*$f_{value},\;f_{pos},\;l_{value},\;l_{pos})$Parameters01 $f_{value}$: the leftmost sensor data value of an interval for recovering compressed sensor data02 $f_{pos}$: the leftmost absolute position of an interval for recovering compressed sensor data03 $l_{value}$: the rightmost sensor data value of an interval for recovering compressed sensor data04 $l_{pos}$: the leftmost absolute position of an interval for recovering compressed sensor dataGlobal VariablesLocal Variables01 *x*: the absolute position of a lost sensor data to be recovered02 $R_{value}$: the value of a recovered lost sensor data with the absolute position *x*Procedure01 Output $f_{pos},\;f_{value}$02 Set $x=f_{pos}+1$03 while(*x*<*l_pos_*) do04 Set $R_{value}=\frac{l_{value}-f_{value}}{l_{pos}-f_{pos}}\cdot \left(x-f_{pos}\right)+f_{value}$05 Output $x,\;R_{value}$06 Set x=x+107 endwhileEndprocedure

($f_{value},\;f_{pos}\;)$ *and* ($l_{value},\;l_{pos}$) are two known endpoints in the algorithm. These endpoints are two pair of value and absolute position about each of two consecutive compressed sensor data in *DataQueue*. The procedure finds the line equation to pass two endpoints (procedure line 4). If there exist one or more absolute positions between these two endpoints, the sensor data at these absolute positions are those lost in compressions. Therefore, the procedure calculates approximately values at these absolute positions by substituting each of them into the line equation one by one (from procedure line 3 to line 7) and outputting each pair of the values and absolute positions of them (procedure line 5). This procedure is used in the *SensorDataRecovery* procedure introduced in the next section.

### 8.2. Sensor Data Recovery Algorithm

*SensorDataRecovery* is an algorithm that receives data structures (*DataQueue*, *Zones*, *ZoneBpSeq*, *ZoneCompHistory*, and *AuxDataQueue*) and a variable (*Ap*) from the SMEP main via a wireless sensor network and existing networks and decompresses or recovers the compressed and lost sensor data by the SMEP main sensor node during communication failure. In fact, all of the sensor data in each zone of *DataQueue* are the compressed or original ground sensor data finally selected in the same number of compressions. *SensorDataRecovery* algorithm finds absolute positions of remaining sensor data in each zone. At this time, the procedure uses *FindAbsoluteAddress* function and this function finds an absolute address using *DataQueu*, *Zones* and *ZoneBpSeq* arrays, when a sensor data position in *DataQueue* is given. Since how to calculate the absolute address using these arrays has already been introduced in the form of a formula in Theorem 8 in Section 6.2, we omit the detailed procedure about that function in this paper. With these sensor data values and found absolute positions, *SensorDataRecovery* procedure recovers the values of lost sensor data to the interpolated using *SensorDataLineInterplolation* procedure described in the previous section. The more detailed algorithm is presented in Algorithm 6. The procedure, at first, finds the last zone of which the zone value is not zero (procedure line 3). The zones from the zone 0 to the non-zero-valued zone are the target zones for decompression and recovery. The zones with 0 as their value includes the original ground sensor data without any compression. Every sensor data in the 0 valued zones doesn’t need any decompression and recovery at all but it just needs output for its value and absolute position. The algorithm pseudo code about this corresponds to the part from the procedure line 29 to 40. The pseudo codes that executes decompression and recovery for none zero-valued target zones are shown from procedure line 5 to 27. Each zone needs a base sensor data for the first decompression and recovery. The pseudo codes for determination of a base sensor data are shown from the procedure line 6 to 9. The base sensor data in the 0 th zone is *DataQueue[0]* as *S_c,_*_0_ and its absolute address is 0 (procedure line 7), since *S_c,_*_0_ is always selected unconditionally in every compression. Meanwhile, the base sensor data for each target zones except the 0 th zone is the value and absolute position in *ZoneCompHistory* to have in its previous zone the same number of compressions with the target zone (procedure line 8). In order to execute decompression and recovery on *DataQueue* sensor data in each target zone with *DataQueue*, the procedure needs to know boundaries of *DataQueue* space corresponding to the target zone. The lowest boundary is determined by the greatest boundary of the previous target zone. Hence, the procedure determines only the greatest boundary as shown from the procedure line 10 to 13. Then, the procedure proceeds decompression and recovery for all sensor data, repeatedly (from procedure line 15 to 21). During doing this, the procedure finds an absolute position of each new sensor data in *DataQueue* through the *FindAbsolutePosition* function (procedure line 17). Then, the procedure calls *SensorDataLineInterpolation* with the values and absolute positions of two old and new sensor data so that each of lost sensor data between these two sensor data should be recovered to an line interpolated value and its absolute position and outputted (procedure line 18). The line interpolation needs two points. By the way, the last sensor data in each zone becomes one of two points as a partner of a previous or base sensor data but, in the next decompression and recovery, it needs a new partner for the line interpolation. So, the procedure uses as its new partner a sensor data of *ZoneCompHistory* of which the value and absolute position had played a role as a base sensor data for the next zone (from the procedure line 22 to 25). Then, the procedure recovers and outputs the compressed and lost sensor data between the last sensor data and its partner by calling *SensorDataLineInterpolation* (from the procedure line 26 to 29).

**Algorithm 6:**
*SensorDataRecovery(DataQueue*, *Zones*, *ZoneBpSeq*, *ZoneCompHistory*, *Ap, AuxDataQueue)*Parameters01 *DataQueue*, *Zones*, *ZoneBpSeq*, *ZoneCompHistory*, *Ap, AuxDataQueue*: *arrays or variables corresponding to SMEP main algorithm parameters*Global VariablesLocal Variable01 *DQinx*: *an index variable for DataQueue array*02 *ZNMaxDQinx*: *the maximum among indices of DataQueue space corresponding to a zone*03 *CompZoneMax*: *the maximum among indices, js, such that Zones[j] is not zero*04 *CompZoneMaxAp*: *the maximum among absolute positions covered by the CompZoneMax zone*05 *ZNinx*: *an index variable in Zones array*06 *S_p_*, *S_j_*: *the leftmost and rightmost values used to recover lost sensor data in an interval, [p, j]*07 *p, j*: *the leftmost and rightmost absolute position used to recover lost sensor data in an interval, [p, j]*08 *ZNCV*: *a value variable of ZoneCompHistory[zone, Zones[zone]].Value for some zone*09 *ZNBP*: *an absolute position variable of ZoneCompHistory[zone, Zones[zone]].Position for some zone*Procedure01 Set *DQinx* = *0*02 if (*Zones[0]*≠0)03 then Find CompZoneMax=max{ k | Zones[k]≠0 }04  Set *ZNinx* = *0,*
*CompZoneMaxAp* = $\sum_{j}^{j=CompZoneMax}2^{m-j-1}\cdot 2^{Zones\left[j\right]}$05  while(0 ≤ *ZNinx* and *ZNinx* ≤ *CompZoneMax*) do06  if (*ZNinx*=*0*)07  then Set *[S_p_, p]* = *[DataQueue[0], 0]*08  else Set *[S_p_, p]* = *ZoneCompHistory[ZNinx−1, Zones[ZNinx]]*09  endif10  if (*ZNinx = m − 1*)11  then *ZNMaxDQinx* = *DQinx+*12  else *ZNMaxDQinx = DQinx*+ $2^{m-ZNinx-1}$13  endif14  *Set DQinx = DQinx + 1*15  while (*DQinx*<*ZNMaxDQinx*) do16  Set *Sj = DataQueue[DQinx]*17  Set *j = FindAbsoluteAddress(DQinx, Zones, ZoneBpString)*18  *SensorDataLineInterpolation(Sp, p, Sj, j)*19  Set *[Sp, p] = [Sj, j]*20  Set *DQinx = DQinx + 1*21  endwhile22    if (*ZNinx* = *CompZoneMax*)23    then Set *[ZNCV, ZNBP] = ZoneCompHistory[ZNinx, 1]*24    else Set *[ZNCV, ZNBP] = ZoneCompHistory[ZNinx, Zones[ZNinx]]*25    endif26  if (*DQinx=ZNMaxDQinx* and *j≠ZNBP*)27   then *SensorDataLineInterpolation(Sj, j, ZNCV, ZNBP)*28;  else Output *S_j_*, *j*29;  endif30;  endwhile31 endif32 if (*CompZoneMax > 0* and *CompZoneMax < m − 1* and *ZNBP≠CompZoneMaxAp*)33 then *SensorDataLineInterpolation(ZNCV, ZNBP, DataQueue[DQinx + 1],*
*CompZoneMaxAp + 1)*34 else if(*CompZoneMax = m − 1 and ZNBP≠*
*CompZoneMaxAp*)35  then *SensorDataLineInterpolation(ZNCV, ZNBP, AuxDataQueue[1],*
*CompZoneMaxAp + 1)*36  endif37 endif38 if (*CompZoneMaxAp*<*m − 1*)39 then Set *offset* = *Ap-CompZoneMaxAp*40 for each *i* such that *1 ≤ I ≤ offset* do41 Output *DataZone[DQinx+i]*, *CompZoneMaxAp + i*42 endfor43 else Set *offset*=*Ap* – *CompZoneMaxAp*44 for each *i* such that 1 *≤* i *≤ offset do*45 Output *AuxDataQueue[i]*, *CompZoneMaxAp* + *i*46 endfor47 endif48 returnEndprocedure

Since if the zone is the last target zone then the first sensor data of the next zone is a ground sensor, the procedure selects, as a partner of the last sensor data, the sensor data of *ZoneCompHistory* to have the least compression 1 in that zone (procedure line 23). There may be the lost sensor data between the last partner selected from *ZoneCompHistory* and the first ground sensor data in the first 0 valued zone. Accordingly, this recovery process appears from the procedure line 32 to 37. This process is not necessary in the cases that the last zero-valued zone is the first zone 0 or the last partner selected from *ZoneCompHistory* is the greatest absolute positioned sensor data covered by the last target zone for decompression and recovery. The processes about these cases are reflected on the procedure lines 32 and 34, respectively. Meanwhile, if the last target zone is the last zone *m − 1* of *DataQueue*, there exists in *AuxDataQueue* a next new partner of the last partner selected from *ZoneCompHistory*, because the new partner does not exist in the next zone any more. If not, however, the new partner exists in *DataQueue*. Each of recovery processes on these cases is reflected on procedure lines 34 and 35 and procedure lines 32, and 33. Furthermore, each case is involved in the next process for ground sensor data in the zero valued zones and *AuxDataQueue*. In other words, in the case that the last target is not the last zone of *DataQueue*, the procedure just outputs each remaining sensor data in *DataQueue* with its absolute position (from the procedure line 38 to 42) since all of ground sensor data remain in *DataQueue*,. Otherwise, the procedure outputs each remaining sensor data in *AuxDataQueue* with its absolute position (from procedure line 38 to 42). Because ground sensor data do not exist in *DataQueue*, any more.

## 9. Performance Comparisons and Analysis

Until now, we have focused on minimizing error for the lost sensor data in compression intervals rather than on minimizing energy consumption, as have most of the current research works. Therefore, our performance evaluation focuses on the average error rate per recovered sensor data as one of performance factors. This section shows average error rates by experimental results based on real-field sensor data sets.

### 9.1. Experimental Environments and Evaluation Measure

#### 9.1.1. Experimental Sensor Data Sets and Samples

Samples extracted from each of four real-field sensor data sets: underwater pH in the ocean, underwater temperature in the ocean, relative humidity in a city, and air temperature in a city. The underwater pH and underwater temperature sets were collected through a wireless sensor network developed by us from a real field: a littoral sea near Yokjido, which is a small island at Tongyeong-Si, Gyeongsangnam-do, Korea. The other sets, relative humidity and air temperature, were collected in Seoul, Korea, by the Meteorological Administration. Each sensor data in each of these four sets is a data collected every alternative hour.

In fact, before choosing these four sensor data sets, we had considered two types of sensor data sets as experimental sensor data sets: one is the type of sensor data set in which changes in the difference among sensor data values are frequent and high. The other is the type of sensor data set in which changes in the difference among sensor data values are infrequent and low. The reason for our consideration is that we had predicted higher error rates in sets with frequent and high value changes than in sets with infrequent and low value changes. Moreover, another reason was to ensure the validity of the average error rate of the SMEP method in sets with frequent and high value changes. Specifically, our main concerns are whether the average error rate in the SMEP method is valid in sets with frequent and high value changes, and until how many times compression is generally reasonable. Two sensor data sets, the relative humidity set and air temperature set, have more frequent and higher value changes than the two remaining sets, the underwater pH set and underwater temperature set. We chose different samples from these four sensor data sets, as shown in Table 2, depending on various experiments. How and why to choose them is described in detail in Section 9.2.1 and Section 9.2.2.

#### 9.1.2. Comparison Target Methods

For comparison with other methods, we have chosen five compression methods, i.e., *winavg*, *delta*, *CQP*, *2MC*, and SMEP. We have applied their own methods to each sample in compression. We have applied a simple line interpolation to decompression and recovery in the SMEP method. We have not applied other interpolations such as two- or three-point spline interpolation, since we had used these interpolation methods but their average error rates were higher in each sample than average error rates by the line interpolation.

#### 9.1.3. Performance Evaluation Measure

For performance evaluation, we use the average error rate defined as the below:

**Definition** **22.** *Let M and* $S^{0}=S_{o,o},S_{0,1},\;\cdots ,\;S_{0,n-1}$ *be a compression method and a ground sensor data sequence, respectively. Given a compressed sensor data sequence of* $S_{0}$*,* $S^{c}=S_{c,o},S_{c,1},\;\cdots ,\;S_{c,m}$*, let* $S^{0^{\prime }}=S_{0,0}^{\prime },\;S_{0,1}^{\prime },\;\cdots ,\;S_{0,n-1}^{\prime }$ *be a decompressed and recovered sensor data sequence of* $S_{c}$. *Then, the average error rate (AER)* $E_{M,S^{c}}$ *of* $S^{c}$ *with respect to* $S^{0}$ *in the compression method M is defined as*

$$E_{M,S_{c}}=\frac{\frac{\sum_{i=1}^{i=n}\left|S_{0,i}^{\prime }-S_{0,i}\right|}{n}}{\frac{\sum_{i=1}^{i=n}\left|S_{0,i}\right|}{n}}=\frac{\sum_{i=1}^{i=n}\left|S_{0,i}^{\prime }-S_{0,i}\right|}{\sum_{i=1}^{i=n}\left|S_{0,i}\right|}$$

From the above definition, note that an average error rate means an average ratio of the average difference value between the recovered sensor data and the original sensor data with respect to the average original sensor data value.

#### 9.1.4. Experimental Tool and Method

We have used MATLAB R2014a as an experimental tool. With this tool, we have experimented with four compression methods per sample, while increasing the number of compressions one-by-one. With our experimental results, we have analyzed and evaluated our SMEP method, comparing it with other methods.

### 9.2. Experiments, Experimental Results and Analysis

In wireless sensor network literatures only a few methods for reducing the sensor data loss during communication failure have been proposed while a lot of the methods for reducing the number of sensor data transmissions with energy efficiency purpose have been mostly presented. They are *winavg*, *delta*, *CQP* and *2MC* methods as they have been briefly discussed in Section 2 and among the the *winavg* and *delta* methods are other’s works. In this section we show experimental results comparing the SMEP method with *winavg*, *delta*, *CQP* and *2MC* methods.

We performed experiments for two categories of analysis: the AER analysis in round compressions and the AER analysis in *Zones* value patterns. In Section 9.2.1 and Section 9.2.2, we present the above two categories of experiments, their experimental results, and the analysis on them, respectively. In Section 9.2.2, moreover, we analyze characteristics of the SMEP method presenting graphs of *Zones* value patterns.

#### 9.2.1. Average Error Rates in Round Compressions

We experimented with four samples for each of the five compression methods, which are *winavg*, *delta*, *CQP*, *2MC*, and *SMEP*. We constructed one sample from each of four real-field sensor data sets, which are relative humidity, air temperature, underwater pH, and underwater temperature sensor data sets. Each sample consisted of 129 consecutive ground sensor data extracted from their corresponding sensor data set. Varying the number of round compressions between one and four for each of four samples per method, we estimated individual AERs and we compared them for the five methods (here, the sizes of original sample in round compressions from one to four times are reduced to 50%, 25%, 12.5%, and 6.25% in compression ratios, respectively). In Table 3, we present the results.

Compared with the other methods, the SMEP shows for each round compressions better AER than the other methods, in the sensor data samples with more frequent and higher value changes, i.e., in the relative humidity and air temperature samples.

In the underwater pH and underwater temperature samples with infrequent and low value changes, though the differences among the AERs of five methods in the same round compressions are small, the SMEP shows mostly better AER than the other methods. Specifically, comparing with the *2MC*, note that the AER of the SMEP method is better than the *2MC*. Meanwhile, in Table 2 the SMEP and the *2MC* show that their AER differences between these methods and the other methods are much bigger in the relative humidity and air temperature samples than in the underwater pH and underwater temperature samples. As an example, in Table 2, while the AER difference between the SMEP and *delta* in the 3rd round compression of the underwater temperature sample is only 0.37%, while their AER difference in the same round compression of the air temperature sample is 21.44%. Therefore, in compressions of ground sensor data with more frequent and higher value changes, the SMEP and *2MC* methods are more effective than the other methods.

We can show that the number of round compressions until which a method is feasible depends definitely on the sensor data change properties in all the compression methods. As shown in Table 2, in all methods, the underwater pH and underwater temperature samples with infrequent and low value changes show the AER difference between the 4th round compression and each of the other round compressions is so small that we can generally ignore it. In contrast to this, every method shows the 4th round compression is not feasible in the relative humidity and air temperature samples with frequent and high value changes. Of course, even if the maximum feasible number of round compressions depends on sensor data properties and sample periods, in these cases, Table 2 shows that the SMEP method is generally feasible up to 3rd round compressions in the case of the samples with frequent and high value changes. In fact, the maximum feasible number of round compressions depends entirely on how finely or frequently sensor data are sampled. The more frequently sampled sensor data are, the higher the maximum feasible number of round compressions is.

#### 9.2.2. Average Error Rates in *Zones* Value Patterns

One among major merits of the SMEP method is *Zones* compression. Such a merit is based on *Zones* rules, in which the numbers of compressions can be different among zones of *DataQueue*. In fact, the round compressions in the experiments described in Section 9.2.1 are the special cases in which values of the non-zero value zones are all the same and there is no ground sensor data in zero-value zones. Hence, these cases are rare. The cases of *Zones* value patterns introduced in this section are much more general and frequent than the cases of round compressions. From now on, we let *m*-*n*-*o*-*p Zones* value pattern or, shortly, *m*-*n*-*o*-*p Zones pattern* mean that values of *Zones[0]*, *Zones[1]*, *Zones[2]*, and *Zones[3]* are *m*, *n*, *o*, and *p*, respectively, in the *DataQueue[0..16]*. In other words, *m*-*n*-*o*-*p Zones pattern* means that sensor data corresponding to *Zones[0]*, *Zones[1]*, *Zones[2]*, and *Zones[3]* in *DataQueue[0..16]* are compressed *m*, *n*, *o*, and *p* times, respectively.

We tried to carry out experiments for investigating the merits in the virtue of *Zones* patterns. For doing these, we prepared *DataQueue[0..16]*. Actually, the *DataQueue* size is much bigger than this size because the *DataQueue* size can be the space size capable of accommodating $2^{m}+1$ sensor data from available free memory space. The real sizes of *DataQueue* in current technologies can generally range from dozens of kilobytes to hundreds of megabytes in available flash memory spaces. Despite this, the reason why we prepared such a small-sized *DataQueue* is to validate the merits of the SMEP method in *Zones* compressions, just with small *DataQueue* size. We also prepared five *Zones* patterns, i.e., 1-0-0-0, 2-0-0-0, 3-0-0-0, 3-2-2-1, and 3-2-2-2, by randomly choosing them because there are too many cases to investigate all cases in *Zones* patterns. The sample sizes corresponding to these patterns are 25, 41, 73, 93, and 97 in order that the ground sensor data should be full in each 0-valued zone. We experimented with five samples with these sizes for each of the five compression methods, which are *winavg*, *delta*, *CQP*, *2MC*, and *SMEP*. Five samples are constructed for each of the four real-field sensor data sets, which are the relative humidity, air temperature, underwater pH, and underwater temperature sensor data sets. According to sample sizes corresponding to the five *Zones* patterns, each of the five samples consisted of consecutive ground sensor data extracted randomly from each of these four sensor data sets. With these samples corresponding to five *Zones* patterns, we estimated the individual AERs and compared them for the five methods. In Table 4, we present the results.

**Figure 12:**
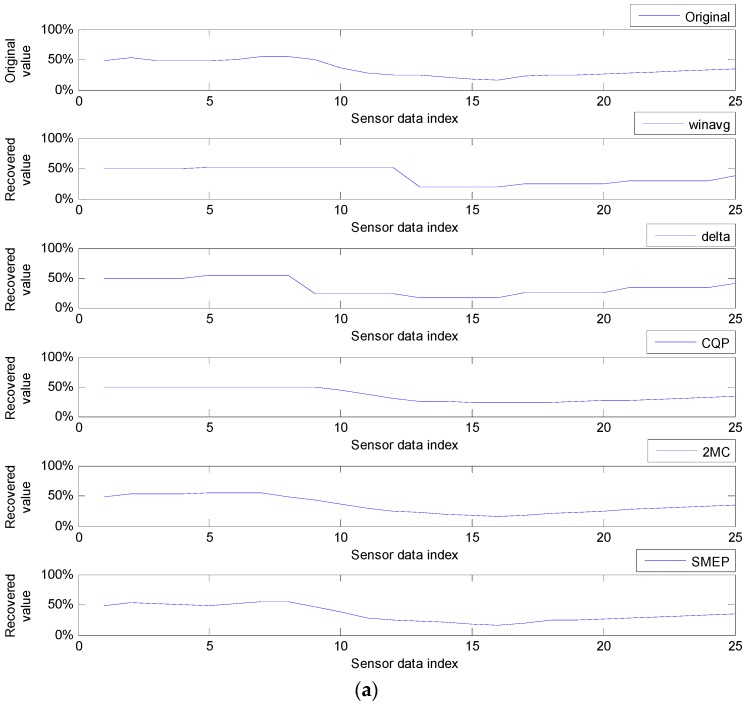

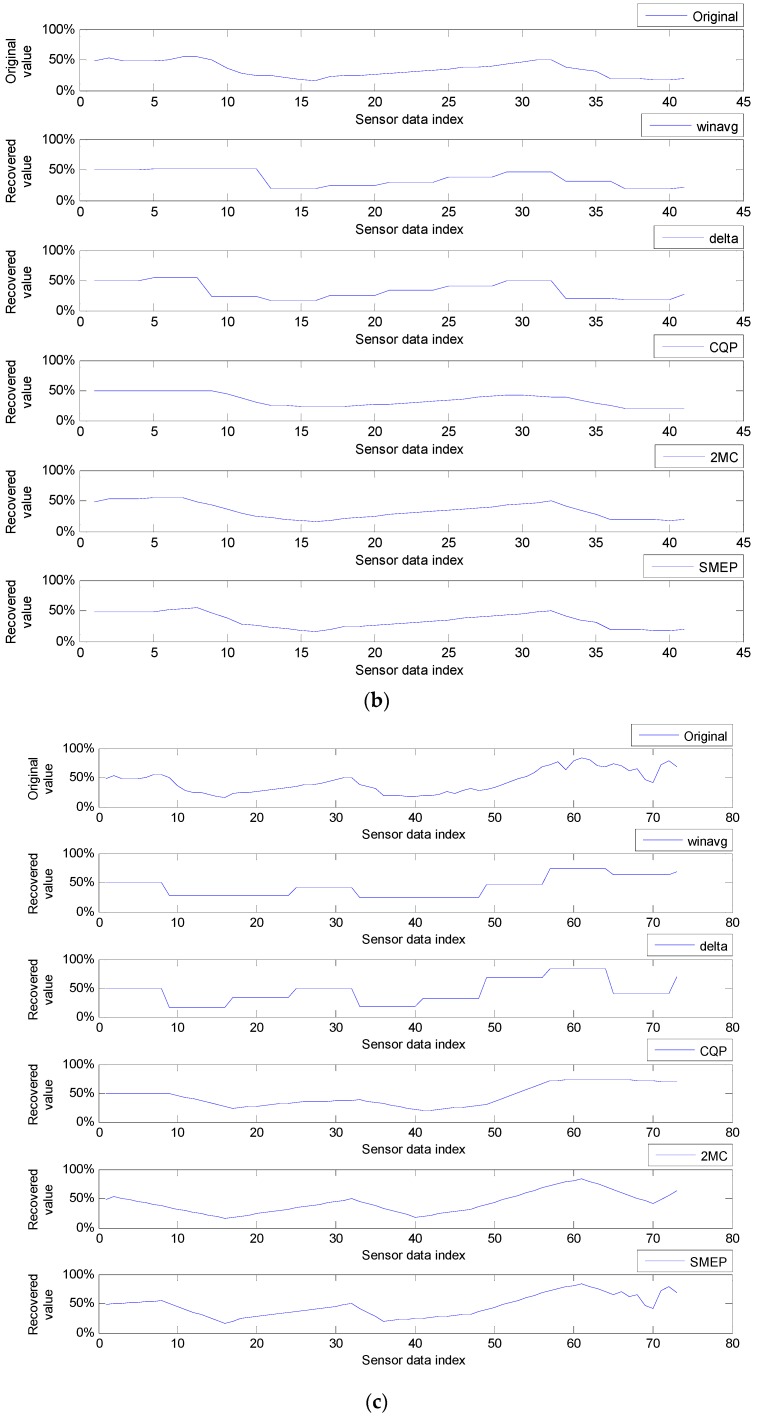

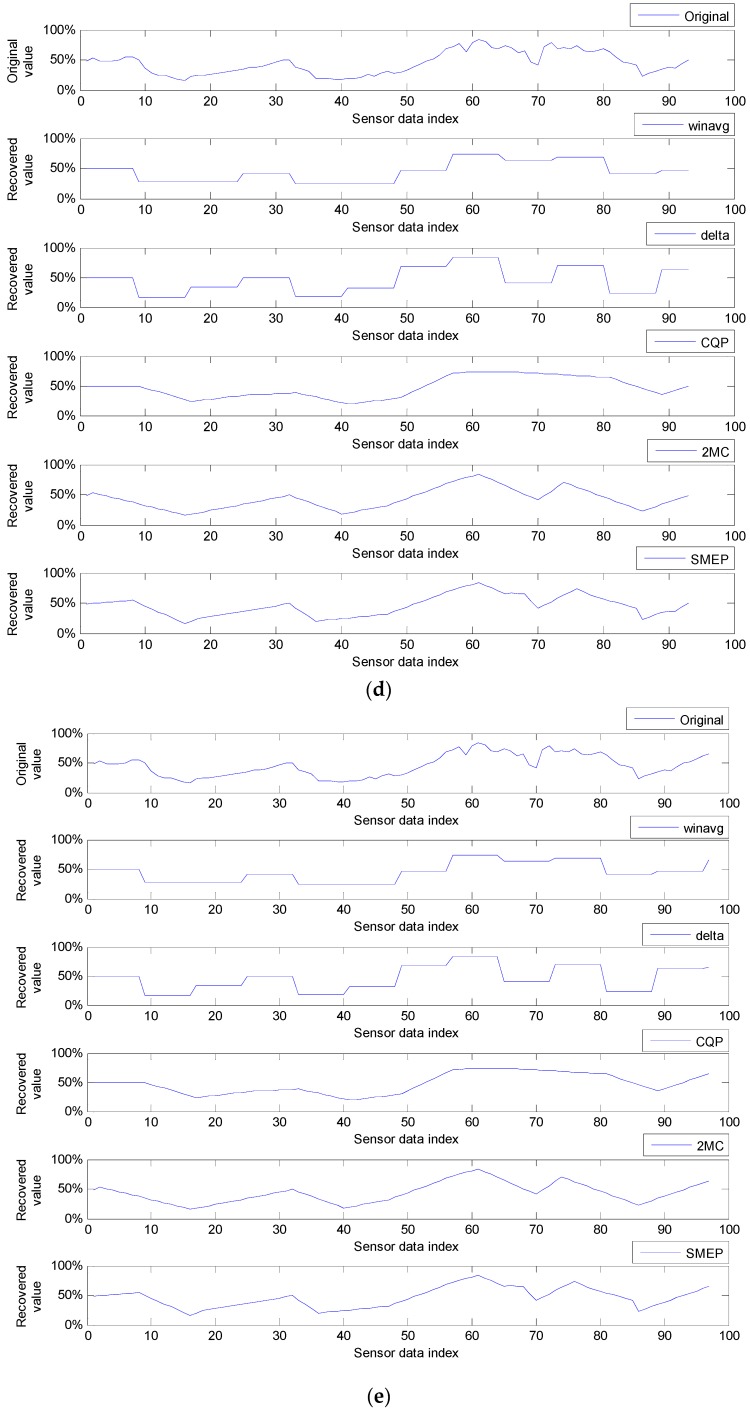
Comparison of graph shape changes in accordance with *Zones* patterns in relative humidity. (**a**) *Zones* pattern: 1-0-0-0; (**b**) *Zones* pattern: 2-0-0-0; (**c**) *Zones* pattern: 3-0-0-0; (**d**) *Zones* pattern: 3-2-2-1**;** (**e**) *Zones* pattern: 3-2-2-2.

Table 4 shows us that even in terms of the zones value patterns, the AER of the SMEP method is better than the AERs of the other methods, too. Figure 12 shows more explicitly the graph shape changes in accordance with *Zones* patterns. The figure shows not only graph patterns of sensor data recovered from relative humidity sensor data compressed by the *winavg*, *delta*, *CQP*, *2MC*, and *SMEP* methods, but also comparisons among between of these patterns and the original sensor data pattern.

In the 1-0-0-0 *Zones* pattern, sensor data from the 1st to the 17th covered by *Zones[0]* are one-time compressed data, but sensor data from the 17th sensor data to the 25th sensor data covered by *Zones[1]*, *Zones[2]*, and *Zones[3]*, of which the values are 0, are ground sensor data. Figure 12a shows the 1-0-0-0 *Zones* pattern. In this graph, we can see that the *CQP*, *2MC*, and SMEP methods follow patterns similar to original sensor data.

Figure 12b shows the 2-0-0-0 *Zones* pattern. In the 2-0-0-0 *Zones* pattern, sensor data between the 1st sensor data and the 33th sensor data are two-time compressed data covered by *Zones[0]*, but sensor data between the 34th sensor data and the 41th sensor data are original sensor data. Like the 1-0-0-0 *Zones* pattern, the *CQP*, *2MC*, and SMEP methods follow patterns similar to original sensor data. Nonetheless, unlike the other methods, including *CQP* and *2MC*, the SMEP method shows that part of the graph between the 34th sensor data and the 41th sensor data is the same pattern as in the original sensor data graph. Actually, this graph part indicates that the SMEP method has a better AER between the original sensor data and its own sensor data part than the other methods.

In the 3-0-0-0 *Zones* pattern, *Zones[0]* sensor data between the 1st sensor data and the 65th sensor data and the sensor data between the 66th sensor data and the 73th sensor data are original sensor data covered by *Zones[1]*, *Zones[2]*, and *Zones[3]*. Figure 12c shows that the graph part covered by *Zones[0]* is more similar to the corresponding original sensor data part than for the other methods. In particular, the graph part covered by *Zones[1]*, *Zones[2]*, and *Zones[3]* is exactly the same as the corresponding original sensor data part.

In the 3-2-2-1 and 3-2-2-2 *Zones* patterns, Figure 12d,e show that graph shapes of the SMEP method are much more similar to the graph shape of the original sensor data than for the other methods. In fact, the graph shape becomes more similar to the original sensor data than the other methods if the *Zones[0]* value is greater than the values of the next non-zero value zones.

One of the reasons for this, as shown in Section 9.2.1, is that the SMEP method generally has a better AER performance than the other methods. Moreover, the more important reason is that in the SMEP method, the number of compressions of sensor data in one zone is different from that in the other zones if the *Zones[0]* value is greater than the values of the next non-zero value zones. In the other methods, however, every sensor data has the same number of compressions as *Zones[0]*. Thereby, the SMEP method tends to exhibit better AER performance effects in the zones with the lower number of compressions than that in *Zones[0]*.

## 10. Conclusions

In this paper, we have proposed a communication framework and feasible method for reducing sensor data that can be lost during communication failure. Our formal approach has also come up with a theoretical basis for problems on the reduction of sensor data loss during communication failure. Consequently, in the comparison with current compression methods, the SMEP method has shown better performance in average error rate per sensor data than others.

Meanwhile, the current technologies have developed and commercialized such cheap micro controller units with flash memories into which program code or data can be permanently saved. Moreover, they are currently used in various wireless sensor network applications. For example, the ATmega128 and Cortex M3 have 128 KB and 512 KB flash memory, respectively. We can use not all of these memory spaces but some parts of them. Under such a possibility, the point is that most current sensor nodes can permanently save compressed and ground sensor data to its own flash memory. Accordingly, our proposed SMEP method is feasible in most of current sensor node technologies. Nonetheless, most sensor data will be lost during communication failures without taking any action.

In the implementation of the SMEP method, the SMEP functionalities cannot be loaded into the routing layer in the protocol stack, because sensor data compression is not the intrinsic function of the routing layer and, in the SMEP method, each compressed or ground sensor data is necessary to be handled not as a packet with extra information, such as a header or footer, but as bare data without any extra information. For these reasons, we recommend that the SMEP functionalities should be embedded into an upper layer of the routing layer. Several research works have introduced and implemented a kind of database layer, such as a query layer [23,34,35,36,45]. In these, the database layer commonly plays a role of data management between the routing layer and upper layers, such as application layer. For the detail on the data layer, refer to the above-referenced literature. Such a database layer or query layer could be one of our recommended upper layers. Another possibility for implementation could be a cross layer to merge the functionalities of the database and routing layers, of course.

On the other hand, the SMEP method alternately applies the *selection by pattern* rule and the *selection by minimum error* rule for each compression interval. Therefore, given two consecutive compression intervals, one is applied by the *selection by pattern* rule and the other is applied by the *selection by minimum error* rule. Now, we expect that we can improve the average error rate if the *selection by minimum error* rule is applied to not one compression interval but several consecutive compression intervals. As further work, we are going to research not only how to design this method, but also how many consecutive compression intervals are optimal for performance and how much its performance is improved depending on the number of consecutive compression intervals.

## Figures and Tables

**Table 1:** Notations and terms.

Notations	Terms	Definitions
*S*	*sensor data sequence*	Definition 1
*S_i_*	*i*-th *sensor data of S*	
*-*	*ground sequence (ground data sequence)*	Definition 2
*-*	*base sequence*	Definition 3
*I = S_i_,S_i+_*_1_*,…,S_i+m_ = [i,i + 1 …,i + m]*	*compression interval*	Definition 4
*-*	*consecutive compression intervals*	Definition 5
*I* ● *J*	*merged compression interval* of *I* and *J*	Definition 6
*C_S_* = *(I*_0_, *I*_1_, *I*_2_, …, *I_m-_*_1_)	*compression interval covering* on *S*	Definition 7
*I_i_*	*i*-th *compression interval in C_S_*	
*-*	*round compression*	Definition 8
*S^c^*	*sensor data sequence with the c-th round compression*	-
*S_c,i_*	*i*-th *sensor data in S^c^*	-
*I_c,i_ (= [p, q]_c_ = [2i,2i + 1]_c_)*	*i*-th *2-size compression interval in C_s_^c^*	-
*-*	*covers*	Definition 9, Definition 10
*I-bit position(*$S_{c,i}$*)*	*I-bit position* of $S_{c,i}$	Definition 11
$B_{p}$	*I-bit position sequence of S*	Definition 12
*BitSubstring(B_p_, c, k)*	*bit substring from the c(k − 1) th bit to ck-1 th bit in B_p_*	Definition 13
*-*	*absolute position of S_c,k_*	Definition 14
*-*	*selection by compression interval pattern with respect to I_c,i_*	Definition 15
*-*	*selection error of a sensor data*	Definition 16
$E_{p}$	*line interpolation errors of* $S_{p}$	Definition 17
*-*	*selection by minimum error rule*	Definition 18
*Zones[0..m − 1]*	*Zones with respect to the data queue DataQueue[0..2^m^]*	Definition 19
*-*	*operations on* Zone*[0..m-1]*	Definition 20
*ZoneBpSeq[0..m − 1]*	*zone bit sequence array*	Definition 21
$E_{M,S^{c}}$	*average error rate (AER) of* $S^{c}$ *with respect to* $S^{0}$ *in the compression method M*	Definition 22

**Table 2:** Experimental environments and performance evaluation measure.

Categories	Experimental Environments and Performance Evaluation Measure
Sensor Data Sets	Relative Humidity, Air Temperature, Underwater pH, Underwater Tmperature
Samples and Sizes	consecutive 129 sensor data sequence extracted from each set, 25, 41, 73, 93 and 97 consecutive sensor data corresponding to 1-0-0-0, 2-0-0-0, 3-0-0-0, 3-2-2-1, and 3-0-0-0 *Zones* patterns, respectively, where *m-n-o-p Zones* pattern means *Zones[0]=m*, *Zones[1]=n*, *Zones[2]=o*, and *Zones[3]* *= p*.
Experimental Tool	MATLAB R2014a
Recovery Method	linear interpolation
Comparison Targets	*winavg*, *delta*, *CQP, 2MC*, and *SMEP* methods
Evaluation Measure	the average error rate (AER) $E_{M,S_{c}}=\frac{\sum_{i=1}^{i=n}\left|S_{0,i}^{\prime }-S_{0,i}\right|}{\sum_{i=1}^{i=n}\left|S_{0,i}\right|}$

**Table 3:** Average error rate (%) for each sensor data set in round compressions (CR: Compression Ratio (%)).

**Sensor Data Sets**		**Relative Humidity**				**Air Temperature**			
	Num. of Round Comps.	1 CR: 50%	2 CR: 25%	3 CR: 12.5%	4 CR: 6.25%	1 CR: 50%	2 CR: 25%	3 CR: 12.5%	4 CR: 6.25%
Methods									
*winavg*		4.60%	9.93%	14.30%	21.35%	5.70%	10.05%	19.66%	31.10%
*delta*		4.62%	12.61%	24.03%	38.40%	5.70%	15.29%	27.27%	50.44%
*CQP*		3.23%	6.57%	10.75%	21.57%	2.11%	4.55%	14.80%	34.02%
*2MC*		3.32%	7.43%	12.15%	25.25%	1.76%	4.39%	6.34%	29.99%
*SMEP*		2.80%	5.72%	10.75%	17.66%	1.24%	3.92%	5.83%	31.94%
**Sensor Data Sets**		**Underwater pH**				**Underwater Temperature**			
	Num. of Round Comps	1 CR: 50%	2 CR: 25%	3 CR: 12.5%	4 CR: 6.25%	1 CR: 50%	2 CR: 25%	3 CR: 12.5%	4 CR: 6.25%
Methods									
*winavg*		0.46%	0.62%	0.82%	0.84%	0.22%	0.31%	0.44%	0.59%
*delta*		0.49%	0.94%	1.83%	3.39%	0.22%	0.51%	0.82%	1.44%
*CQP*		0.36%	0.68%	1.09%	1.84%	0.20%	0.34%	0.53%	0.71%
*2MC*		0.29%	0.44%	0.68%	0.81%	0.17%	0.34%	0.45%	0.82%
*SMEP*		0.24%	0.39%	0.62%	0.62%	0.16%	0.32%	0.45%	0.69%

**Table 4:** Average error rates in sensor data samples corresponding to *Zones* value patterns (*DataQueue* size: 16, CR: Compression Ratio (%)).

**Sensor Data Sets**		**Relative Humidity**					**Air Temperature**				
	*Zones*	1-0-0-0 CR: 67%	2-0-0-0 CR: 40%	3-0-0-0 CR: 22%	3-2-2-1 CR: 17%	3-2-2-2 CR:16.7%	1-0-0-0 CR: 67%	2-0-0-0 CR: 40%	3-0-0-0 CR: 22%	3-2-2-1 CR: 17%	3-2-2-2 CR: 16.7%
Methods											
*winavg*		4.36%	11.03%	14.42%	14.36%	14.30%	5.78%	12.67%	20.64%	19.67%	19.66%
*Delta*		4.62%	11.93%	24.10%	25.10%	24.03%	5.93%	17.78%	28.29%	28.47%	27.27%
*CQP*		2.59%	6.71%	11.55%	11.27%	10.75%	2.40%	6.22%	16.70%	15.27%	14.80%
*2MC*		2.35%	5.15%	10.99%	12.05%	11.50%	1.65%	5.56%	6.97%	6.26%	6.08%
*SMEP*		2.10%	2.57%	7.75%	8.71%	8.64%	1.36%	3.64%	5.86%	5.42%	5.25%
**Sensor Data Sets**		**Underwater pH**					**Underwater Temperature**				
	*Zones*	1-0-0-0 CR: 67%	2-0-0-0 CR: 40%	3-0-0-0 CR: 22%	3-2-2-1 CR: 17%	3-2-2-2 CR:16.7%	1-0-0-0 CR: 67%	2-0-0-0 CR: 40%	3-0-0-0 CR: 22%	3-2-2-1 CR: 17%	3-2-2-2 CR: 16.7%
Methods											
*winavg*		1.29%	1.20%	0.96%	0.86%	0.82%	0.25%	0.32%	0.50%	0.45%	0.44%
*Delta*		1.35%	1.95%	2.30%	1.91%	1.83%	0.27%	0.53%	0.91%	0.83%	0.82%
*CQP*		0.90%	1.25%	1.35%	1.14%	1.09%	0.34%	0.43%	0.61%	0.53%	0.53%
*2MC*		0.48%	0.70%	0.83%	0.74%	0.71%	0.28%	0.36%	0.52%	0.46%	0.46%
*SMEP*		0.36%	0.61%	0.68%	0.62%	0.64%	0.22%	0.36%	0.49%	0.45%	0.44%

m-n-o-p *Zones* pattern means *Zones[0]* *=* m, *Zones[1]* *=* n, *Zones[2]* *=* o, and *Zones[3]* *=* p.

## Appendix A. Proof of Theorem 1

**Proof.** Since *I* = [*i*, *j*] and *J* = [*k*, *l*] are interval *i* < *j* and *k* < *l*. Moreover, since compression intervals *I* and *J* are consecutive, either *j* = *k* or *l* = *i* is true. But, if *l* = *i*-th*en k < l = i < j* and this is a contradiction to the conditions of the theorem, *i* < *k* or *i* < *l* or *j* < *l*. Thus, *j* = *k* is true. Therefore, by the definition of consecutive compression interval, *S_i_*, *S_i+_*_1_, …, *S_j−_*_1_, *S_j_* and *S_k_*, …, *S_l−_*_1_, *S_l_* are consecutive sensor data, respectively, and since *S_j_* = *S_k_* = *S_i+m_*, [*i*, *l*] = *S_i_*, *S_i+_*_1_, …, *S_j+m−_*_1_, *S_j_*, *S_k+_*_1_, …, *S_l+_*_1_, *S_l_* are success sensor data and the number of them is *n+ m*. By definition, *J* = [*i*, *l*] is a compression interval with the size *m + n* with respect to *S*. ☐

## Appendix B. Proof of Theorem 3

**Proof** **of Proposition 1.** In initial step, if m = 1, *C_S_* = *(I*_0_*)* and *I*_0_ = *S*. Thus, |*I*_0_|=|*S*|=3=2⋅1+1. So, when m=0, *Proposition 1* is true. Assume that the proposition is true for an integer *k*, *1<k< m*. For *k=m*, |*S*| = |*I*_0_ ● *I*_1_ ● *I*_2_ ● … ● *I_m_*| = |(*I*_0_ ● *I*_1_ ● *I*_2_ ● … ● *I_m−_*_1_) ● *I_m−_*_1_| (by Theorem 2) = |*I*_0_ ● *I*_1_ ● *I*_2_ ● … ● *I_m−_*_1_|+|*I_m_*| (by Theorem 1) = *(2(m − 1)+1)+2 (since |I*_0_ *● I*_1_ *● I*_2_ *● …* *● I_m−_*_1_*| = 2(m − 1)+1 by the assumption) = 2m + 1*. By induction, *Proposition 1* is true. ☐

**Proof** **of Proposition 2.** For *p, q, r* of *S_p_, S_q_, S_r_* in *I_i_*, *q = p + 1* and *r = q + 1 = p + 2*, since they are consecutive in a compression interval. *p = 0* for *i = 0* since *I*_0_ is the first compression interval and *S_p_* is the first sensor data and so, *p = 0 = 2**⋅0*, *q = 1 = 2**⋅0 + 1*, *r = 2 = 2**⋅0 + 2*. Thus, *Proposition 2* is true for *i = 0*. Assume that the proposition is true for *0 < k < i*. For *k* = *i* − 1, the position of the last sensor data in the previous compression interval *I_i−_*_1_ is *2(I − 1) + 2* (by the assumption) = *2i*. Since *I_i−_*_1_ and *I_i_* are consecutive compression intervals, the last sensor data in *I_i_* is shared as the same one as the first sensor data in *I_i_*. Thus*, p = 2i*. Consequently, *q = 2i + 1* and *r = 2i + 2*. Thus, by induction, *Proposition 2* is true for an integer, i, *0 ≤ i ≤ m − 1*. ☐

## Appendix C. Proof of Theorem 4

**Proof.** Let the size of *S* be *n*. If *m* = 1, then *n* = 3, *I*_0_ = *S* = *S*_0_, *S*_1_, *S*_2_, *Cs* = (*I*_0_), and *k* = *0* = 2^1*−*1^ − 1. Thus, the theorem is true for *m* = 1. Assume that the theorem is true for any integer *k*, *k* < *m*. If *k*=*m*, the size of *S* is *2^m^ +* 1. So, let *S* = *S*_0_*, S*_1_, *….*, *S*_2_*^m−^*^1^*_−_*_1_*, S*_2_*^m−^*^1^*, S*_2_*^m−^*^1^*_+_*_1_*, …, S*_2_*^m^.* Since *2m = 2**⋅2^m−^*^1^ *= 2^m−^*^1^ *+ 2^m−^*^1^, *S = S*_0_*, S*_1_, …., *S*_2_*^m−^*^1^, *S*_2_*^m−^*^1^*_+_*_1_, …, *S*_2_*^m−^*^1^*_+_*_2_*^m−^*^1^. Now, divide *S* into two same size sensor data sequences, *P = S*_0_*, S*_1_*, …., S*_2_*^m−^*^1^ and *Q = S*_2_*^m−^*^1^*, S*_2_*^m−^*^1^*_+_*_1_*, …, S*_2_*^m−^*^1^*_+_*_2_*^m−^*^1^, respectively, where *S*_2_*^m−^*^1^ is common. Therefore, each size of the two is *2^m−^*^1^ *+ 1*. Consider two 2-size compression intervals, *C_P_ = (J*_0_, *J*_1_, *…*, *J_p_)* and *C_Q_ = (H*_0_, *H*_1_, …, *H_q_)* with respect to *P* and *Q*, respectively. Then, by assumption, *p = q = 2^m−^*^2^ *− 1*, thus number of compression intervals in both of *C_P_* and *C_Q_* is *2^m−^*^2^. Moreover, *(J*_0_, *J*_1_, *…., J_p_, H*_0_, *H*_1_, *…*, *H_q_)* = *C_s_ = (I*_0_, *I*_1_,…, *I_l_)*, and since the number of compression intervals in *C_s_* is *2^m−^*^2^ *+ 2^m−^*^2^ *= 2^m−^*^1^, *l = 2^m−^*^1^ *− 1* and the theorem has been proved. ☐

## Appendix D. Proof of Theorem 5

**Proof.** We prove the theorem by induction. At first, for *k = 1*, *S*_1*,i*_ covers *I*_0*,i−*1_ = *I*_0*,(i−*1*)*2_*°* = *I_k,(i−_*_1*)*2_*^k−^*^1^ by Lemma 1. Assume that the theorem is true for integer *k ≤ c*. By Lemma 2, *S_c,i_* covers *I_c−_*_1*,i−*1_ and *I_c−_*_1*,i−*1_ covers *I_c−_*_2*,*2*(i−*1*)*_●*I_c−_*_2*,*2*i−*1_. By assumption, *I_c−_*_2*,*2*(i−*1*)*_ covers *I*_0*,*2_*_⋅_*_2*(i−*1*)*2_*^c−^*^3^ ● *I*_0*,*2_*_⋅_*_2*(i−*1*)*2_*^c−^*^3^*_+_*_1_ ● ⋅⋅⋅ ● *I*_0*,*2_*_⋅(_*_2*(i−*1*)+*1*)*2_*^c−^*^3^*_−_*_1_ = *I*_0*,(i−*1*)*2_*^c−^*^1^ ● *I*_0*,(i−*1*)*2_*^c−^*^1^*_+_*_1_ ● ⋅⋅⋅ ● *I*_0*,(*2*i−*1*)*2_*^c−^*^2^*_−_*_1_ and *I_c−_*_2*,*2*i−*1_ covers *I*_0*,*2*(*2*i−*1*)*2_*^c−^*^3^ ● *I*_0*,*2*(*2*i−*1*)*2_*^c−^*^3^*_+_*_1_ ● ⋅⋅⋅ ● *I*_0*,*2_*_⋅_*_2*i*_*_⋅_*_2_*^c−^*^3^*_−_*_1_ = *I*_0*,(*2*i−*1*)*2_*^c−^*^2^ ● *I*_0*,(*2*i−*1*)*2_*^c−^*^2^*_+_*_1_ ● ⋅⋅⋅ ● *I*_0*,i*2_*^c−^*^1^*_−_*_1_. Thus, *S_c,i_* covers *I_c−_*_2*,*2*(i−*1*)*_●*I_c−_*_2*,*2*i−*1_ = *I*_0*,(i−*1*)*2_*^c−^*^1^ ● *I*_0*,(i−*1*)*2_*^c−^*^1^*_+_*_1_ ● ⋅⋅⋅ ● *I*_0*,(*2*i−*1*)*2_*^c−^*^2^*_−_*_1_ ● *I*_0*,(*2*i−*1*)*2_*^c−^*^2^ ● *I*_0*,(*2*i−*1*)*2_*^c−^*^2^*_+_*_1_ ● ⋅⋅⋅ ● *I*_0*,i*2_*^c−^*^1^*_−_*_1_ and the theorem has been proved. ☐

## Appendix E. Proof of Theorem 7

**Proof.** Since *p* = 4*k* + 1 and *q* = 4*k* + 2, by definition,

$$\begin{array}{c} E_{p}=E_{4k+1}=\left|S_{c,q}-A\;\right|=\left|S_{c,4k+2}-A\right|=\left|S_{c,4k+2}-\left(\frac{S_{c,j}-S_{c,4k+1}}{j^{\prime }-p^{\prime }}\left(q^{\prime }-p^{\prime }\right)+S_{c,4k+1}\right)\;\right|\\ =\left|-\;\frac{S_{c,j}-S_{c,4k+1}}{j^{\prime }-p^{\prime }}\left(q^{\prime }-p^{\prime }\right)+(S_{c,4k+2}-S_{c,4k+1})\right|\\ =\left|\frac{S_{c,j}-S_{c,4k+1}}{j^{\prime }-p^{\prime }}\left(q^{\prime }-p^{\prime }\right)-(S_{c,4k+2}-S_{c,4k+1})\right|\\ \text{and}\\ E_{q}=E_{4k+2}=\left|S_{c,p}-B\;\right|=\left|S_{c,4k+1}-B\right|=\left|S_{c,4k+1}-\left(\frac{S_{c,i}-S_{c,4k+2}}{i^{\prime }-q^{\prime }}\left(p^{\prime }-q^{\prime }\right)+S_{c,4k+2}\right)\;\right|\\ =\left|\frac{S_{c,i}-S_{c,4k+2}}{i^{\prime }-q^{\prime }}\left(q\prime -p^{\prime }\right)-\;(S_{c,4k+2}-S_{c,4k+1})\right| \end{array}$$

☐

## Appendix F. Proof of Theorem 8

**Proof.** In proof of (*i)*, above all, for any *j* such that *0 ≤ j ≤ m − 1* the *DataQueue* size covered by *Zones[j]* is *2^m−j−^*^1^. If *DataQueue[i]* exists in the zone *k*, then $\sum_{j=0}^{j=k-1}2^{m-j-1}<i<\sum_{j=0}^{j=k+1}2^{m-j-1}$ since *k − 1* < *k* < *k+1*. Accordingly, $i-\sum_{j=0}^{j=k-1}2^{m-j-1}>0$ **and** $i-\sum_{j=0}^{j=k+1}2^{m-j-1}<\;0$. Thus, *k* is the maximum number to satisfy $i-\sum_{j=0}^{j=k-1}2^{m-j-1}>0$, this is, $k=max\left\{\;t\;|\;0<i-\sum_{j=0}^{j=t-1}2^{m-j-1}\right\}$. Since $\sum_{j=0}^{j=t-1}2^{m-j-1}=2^{m-1}+2^{m-2}+\ldots +2^{m-t}=2^{m}-2^{m-t}=2^{m-t}\left(2^{t}-1\right)$, $k=max\left\{\;t\;|\;0<\left(i-2^{m-t}\cdot \left(2^{t}-1\right)\right)\right\}$. If *i =* 2*^m^*, *i* is the last position. Hence, *i* exists in the last zone *m − 1*. In the *(ii)* proof, at first, if *i* = 0, *P_i_* = *0*, since *S_c,_*_0_ = *S*_0,0_ for any number of compressions *c*. Now, we prove the case for *i* ≠ *0* and *k* ≥ *0*. Let *k* be the zone in which *i* position is located. The absolute address *P_i_* of *i* position in *DataQueue* consists of a sum of the three parts:

(A1) $$P_{i}=P_{B}+P_{k,\;i-1}+P_{k,I_{i}\;}$$

where $P_{B}$ is the number of absolute positions covered by zones from the zone 0 to the zone *k* − 1, $P_{k,\;i-1}$ is the number of absolute positions within a zone *k* which covered by sensor data from 1 st sensor data to the *i −* 1 th positioned sensor data in the zone, $P_{k,I_{i}}$ is an *I-bit position* address of the *i*-th position within the compression interval in which the *i*-th position is located. Each zone *j* has $2^{m-j-1}$ sensor data in *DataQueue* and each of them has been selected among $2^{Zones\left[j\right]}$. Therefore, each zone *i* covers $2^{m-j-1}\cdot 2^{Zones\left[j\right]}$ absolute positions. Since zones from the zone *0* to the zone *k − 1* are located before the zone *k*,

(A2) $$P_{B}=\sum_{j=0}^{k-1}2^{m-j-1}\cdot 2^{Zones\left[j\right]}$$

Meanwhile, the number of relative positions in the zone *k* for *I − 1* position is $j-1-\sum_{j=0}^{k-1}2^{m-j-1}\cdot 2^{Zones\left[j\right]}$ and each of $j-1-\sum_{j=0}^{k-1}2^{m-j-1}\cdot 2^{Zones\left[j\right]}$ sensor data covers $2^{Zones\left[k\right]}$ in the zone *k* since it has passed through *Zones[k]* compressions. Thus,

(A3) $$P_{k,i-1}=(j-1-\sum_{j=0}^{k-1}2^{m-j-1})\cdot 2^{Zones\left[k\right]}$$

The relative position of the position *i* in the zone *k* is $j-\sum_{j=0}^{k-1}2^{m-j-1}\cdot 2^{Zones\left[j\right]}$. Using this position, the I-bit position sequence *ZoneBpSeq[k]* in the zone *k*, and the *BitSubstring* function,

(A4) $$P_{k,I_{i}\;}={\left(BitSubstring(ZoneBpSeq\left[k\right],\;Zones\left[k\right],\;i-\overset{j=k-1}{\underset{j=0}{\sum }}2^{m-j-1})\right)}_{10}+1$$

Replacing Equation (1) with Equation (2)–(4) finally,

$$\begin{array}{c} P_{i}=P_{B}+P_{k,\;i-1}+P_{k,I_{i}}\\ =\;\sum_{j=0}^{k-1}2^{m-j-1}\cdot 2^{Zones\left[j\right]}+(j-1\;-\sum_{j=0}^{k-1}2^{m-j-1})\cdot 2^{Zones\left[k\right]}+\\ {\left(BitSubstring(ZoneBpSeq\left[k\right],\;Zones\left[k\right],\;i-\sum_{j=0}^{j=k-1}2^{m-j-1})\right)}_{10}+1 \end{array}$$

We have completed the proof of the theorem. ☐

## Appendix G. Proof of Theorem 9

**Proof.** Let *L* be the size of a sensor data sequence from the StartZone zone to the last zone. Then,

$$L=2^{m-StatZone}.$$

Because $L=2^{m}-\mathrm{the}\;\mathrm{number}\;\mathrm{of}\;DataQueue\;\mathrm{positions}\;\mathrm{in}\;\mathrm{zones}\;\mathrm{before}\;\mathrm{the}\;StartZone\;\mathrm{zone}$

$$=2^{m}-\sum_{j=0}^{StartZone-1}2^{m-j-1}=2^{m}-\left(2^{m}-2^{m-StartZone}\right)=2^{m-StartZone}.$$

Given a sensor data sequence with even number size, the sensor data sequence after compression becomes to be reduced to the half size. Thus, the compressed sensor data sequence with the size *L* is reduced to the size $\frac{1}{2}L=\frac{1}{2}\cdot 2^{m-StatZone}=2^{m-StatZone-1}$, which is the same as the size of *StartZone* zone. Thus, the sensor data sequence in consecutive equivalent zones from the *StartZone* zone to the last *m −* 1 zone is compressed to the *Startzone* zone. ☐

## Appendix H. Proof of Lemma 1

**Proof.** By induction, for *k* = 0, the theorem is true because a sensor data selected in *I_c,_*_0_ is the one selected among sensor data selected in *I_c−_*_1*,*0_*=I_c,_*_2_*_⋅_*_0_ and *I_c−_*_1*,*1_ *= I_c,_*_2_*_⋅_*_0*+*1_ so that *I_c,o_* covers *I_c−_*_1*,*2_*_⋅_*_0_●*I_c−_*_1*,*2_*_⋅_*_0*+*1_. Assume that the theorem is true for any integer *i ≤ k − 1*. Consider *I_c−_*_1*,*2*k−*2_, *I_c−_*_1*,*2*k−*1_, *I_c−_*_1*,*2*k*_, and *I_c−_*_1*,*2*k+*1_. They are consecutive depending on the order of compression intervals in *C_s_^c^*. Here, *I_c−_*_1*,*2*k−*2_ = *I_c−_*_1*,*2*(k−*1*)*_ and *I_c−_*_1*,*2*k−*1_ = *I_c−_*_1*,*2*(k−*1*)+*1_, *I_c−_*_1*,*2*k−*2_ and therefore, *I_c,k−_*_1_ covers *I_c−_*_1*,*2*k−*2_ ● *I_c−_*_1*,*2*k−*1_. Thus, the *I_c,k_* must cover *I_c−_*_1*,*2*k*_●*I_c−_*_1*,*2*k+*1_. Consequently, for *i ≤ k − 1*, the theorem is true. ☐

## Appendix I. Proof of Lemma 2

**Proof.** *S_c,i_* is selected in *I_c−_*_1*,i−*1_. Thus, *I_c−_*_1*,i−*1_ = *I_c−_*_1*,j*_ and *j* = *i* − 1. Moreover, since *S_c,i_* covers *I_c−_*_1*,i−*1_, by lemma 1, *I_c−_*_1*,j*_ covers *I_c−_*_2*,*2*(i−*1*)*_●*I_c−_*_2*,*2*i−*1_ = *I_c−_*_2*,*2*(i−*1*)*_●*I_c−_*_2*,*2*(i−*1*)+*1_. ☐

## Appendix J. Proof of Lemma 3

**Proof.** The lemma 3 is trivial. Because the I-bit position of *S_c,k_* is positioned at the *k-*th I-bit position in the I-bit position sequence, the length of I-bit position is c, and I-bit position of *S_c,k_* is located at the c(*k − 1*) th position in the I-bit position sequence (note that the first position in I-bit position sequence is 0). ☐

## Appendix K. Proof of Lemma 4

**Proof.** For *k* = *0*, *S_c,_*_0_ is always selected as a first sensor data unconditionally from *S*_0*,*0_. Therefore, *i* = *0*. For *k* ≥ *1*, according to corollary of Theorem 5, the position of *S_c,k_* exists in [*(k − 1)2^c^*, *k2^c^*]_0_, the k − 1 th compression interval of *S*^0^. Thus, the absolute position of the first sensor data in the interval *[(k − 1)2^c^, k2^c^]*_0_ is *(k − 1)2^c^*. Since the relative position of *S_c,k_* is (*I-bit position(S_c,k_) )*_10_ *+ 1*, its absolute position *i* is *(k − 1)2^c^* + *(I-bit position(S_c,k_))*_10_ + 1. ☐

## Appendix L. Proof Lemma 5

**Proof.** Given *B_p_*, in the definition, we can get the absolute position *i’* and *j’* for *S_c,i_* and *S_c,j_*, respectively, using *B_p_* by the theorem. Moreover, we can get the absolute position *p’* and *q’* for *S_c,p_* and *S_c,q_* in the same way. In fact, the absolute positions of *i’*, *j’*, *p’* and *q’* are

*i’* = *(i − 1)2^c^* + *(BitSubstring(B_p_*, *c*, *i))*_10_, *j’* = *(j − 1)2^c^* + *(BitSubstring(B_p_*, *c*, *j))*_10_, *p’* = *(p − 1)2^c^* + *(BitSubstring(B_p_*, *c*, *p))*_10_, *q’* = *(q − 1)2^c^* + *(BitSubstring(B_p_*, *c*, *q))*_10._

Meanwhile, line equations $\bar{S_{c,p}S_{c,j}}$ and $\bar{S_{c,i}S_{c,q}}$ by using absolute positions are as follows:

(A5) $$\bar{S_{c,p}S_{c,j}}=\;\bar{S_{0,p^{\prime }}S_{0,j^{\prime }}}=\;\frac{S_{o,j^{\prime }}-S_{0,p^{\prime }}}{j^{\prime }-p^{\prime }}\left(x-p^{\prime }\right)+S_{0,p^{\prime \;=\;}}\frac{S_{c,j}-S_{c,p}}{j^{\prime }-p^{\prime }}\left(x-p^{\prime }\right)+S_{c,p}\text{ and }\;\bar{S_{c,i}S_{c,q}}=\;\bar{S_{0,i^{\prime }}S_{0,q^{\prime }}}=\;\frac{S_{o,i^{\prime }}-S_{0,q^{\prime }}}{i^{\prime }-q^{\prime }}\left(x-q^{\prime }\right)+S_{0,q^{\prime \;=\;}}\frac{S_{c,i}-S_{c,q}}{i^{\prime }-q^{\prime }}\left(x-q^{\prime }\right)+S_{c,q},\;\text{since }S_{c,i}=S_{0,i^{\prime \;}},S_{c,j}=S_{0,j^{\prime }},\;S_{c,p}=S_{0,p^{\prime \;}}\text{ and }S_{c,q}=S_{0,q^{\prime }}.$$

Since in Figure 7a,b are the points *q’* and *p’* at on the line $\bar{S_{0,p^{\prime }}S_{0,j^{\prime }}}$ and $\bar{S_{0,i^{\prime }}S_{0,q^{\prime }}}$, respectively, now, we can compute *A* and *B* such that

(A6) $$A=\frac{S_{c,j}-S_{c,p}}{j^{\prime }-p^{\prime }}\left(q\prime -p^{\prime }\right)+S_{c,p}\text{ and }B=\frac{S_{c,i}-S_{c,q}}{i^{\prime }-q^{\prime }}\left(p\prime -q^{\prime }\right)+S_{c,q}$$

By the way, since *Sp* and *Sq* is the second and the third senor data in *I_c,_*_2*k*_, *p* = 4*k* + 1 and *q* = 4*k* + 2, replacing them with *p* and *q* in (Equation (6)), we can get

$$A=\frac{S_{c,j}-S_{c,4k+1}}{j^{\prime }-p^{\prime }}\left(q\prime -p^{\prime }\right)+S_{c,4k+1}$$

$$B=\frac{S_{c,i}-S_{c,4k+2}}{i^{\prime }-q^{\prime }}\left(p\prime -q^{\prime }\right)+S_{c,4k+2}$$

☐
